# Combining Network Pharmacology and Experimental Verification to Ascertain the Mechanism of Action of *Asparagus officinalis* Against the Brain Damage Caused by Fluorosis

**DOI:** 10.1002/tox.24382

**Published:** 2024-07-23

**Authors:** Feiqing Wang, Yang Liu, Yanju Li, Xu Yang, Jianing Zhao, Bo Yang, Dongxin Tang, Chike Zhang, Zhixu He, Dong Ming, Xiaodong Zhu

**Affiliations:** ^1^ Academy of Medical Engineering and Translational Medicine Tianjin University Tianjin China; ^2^ Clinical Research Center The First Affiliated Hospital of Guizhou University of Traditional Chinese Medicine Guiyang Guizhou China; ^3^ Department of Hematology Affiliated Hospital of Guizhou Medical University Guiyang Guizhou China; ^4^ National & Guizhou Joint Engineering Laboratory for Cell Engineering and Biomedicine Technique Guiyang Guizhou China; ^5^ Neurological Institute, Tianjin Medical University General Hospital Tianjin China

**Keywords:** *Asparagus*, brain, fluorosis, network pharmacology, SIRT1/BDNF/TrkB signaling pathway

## Abstract

*Asparagus officinalis* (ASP) has antioxidation, anti‐inflammatory, antiaging, and immune system‐enhancing effects. We explored the preventive and therapeutic consequences of ASP on the brain damage elicited by fluorosis through network pharmacology and in vivo experimental validation. We ascertained the pharmaceutically active ingredients and drug targets of ASP from the Traditional Chinese Medicine Systems Pharmacology database, predicted the disease targets of fluorosis‐induced brain injury using GeneCards and Online Mendelian Inheritance in Man databases, obtained target protein–protein interaction networks in the Search Tool for the Retrieval of Interacting Genes/Proteins database, used Cytoscape to obtain key targets and active ingredients, and conducted enrichment analyses of key targets in the Database for Annotation, Visualization and Integrated Discovery. Enrichment analyses showed that “mitogen‐activated protein kinase” (MAPK), “phosphoinositide 3‐kinase/protein kinase B” (PI3K‐Akt), “nuclear factor‐kappa B” (NF‐κB), and the “neurotrophin signaling pathway” were the most enriched biological processes and signaling pathways. ASP could alleviate fluorosis‐based injury, improve brain‐tissue damage, increase urinary fluoride content, and improve oxidation levels and inflammatory‐factor levels in the body. ASP could also reduce dental fluorosis, bone damage, fluoride concentrations in blood and bone, and accumulation of lipid peroxide. Upon ASP treatment, expression of silent information regulator (SIRT)1, brain‐derived neurotrophic factor (BDNF), tropomyosin receptor kinase B (TrkB), MAPK, NF‐κB, PI3K, Akt, and B‐cell lymphoma‐2 in rat brain tissue increased gradually, whereas that of Bax, caspase‐3, and p53 decreased gradually. We demonstrated that ASP could regulate the brain damage caused by fluorosis through the SIRT1/BDNF/TrkB signaling pathway, and reported the possible part played by ASP in preventing and treating fluorosis.

## 1 Introduction

Fluorine is a vital trace element for human life activities. It is crucial for the development of human bones and teeth. Usually, fluorine exists in the form of compounds in nature. Fluorine is an element, and fluoride is an ion or a compound, which contains the fluoride ion. Long‐term exposure to fluoride in water, air, or food can cause fluorosis. Endemic fluorosis is a global public‐health problem. Epidemiological reports have shown that over 260 million people worldwide are exposed to high levels of fluoride in the environment [1]. China has the highest prevalence of fluorosis. It faces the most serious harmful effects of fluorosis in the world because China is located in the “fluoride belt.” Excess fluoride levels have been detected in the groundwater in areas of China where the fluoride concentration in rocks or soil is high. The earliest pathologic changes wrought by fluorosis are mainly in teeth and bones [2]. In addition to obvious damage to bone tissue, fluorosis causes varying levels of harm to the kidney, liver, and systems (nervous, endocrine, intestinal, cardiovascular, other), and its pathogenesis is the focus of medical research [3, 4, 5].

Fluorine has active chemical properties and strong oxidizing properties, which can induce an increase in the manufacture of reactive oxygen species in cells. The accumulation of fluoride in the body can lead to an increase in the construction of oxygen free radicals. This action leads to oxidative stress (OS) and the creation of many OS products [6, 7, 8]. Fluorosis can cause a reduction in the activity of antioxidant enzymes and/or an increase in lipid‐peroxidation products in the body [9]. There is a mutually promoting relationship between OS and the inflammatory response [10, 11]. The concentrations of oxidative products and inflammatory factors within the body have been employed as markers of the tissue damage caused by fluorosis [12].

Numerous epidemiological and experimental reports have shown that long‐term excessive intake of fluoride into the body can elicit destructive effects on human tissues and organs. Its toxic damage cannot be undone, especially the neurotoxicity caused in children, which leads to a reduction in their intelligence in areas blighted by fluorosis [13, 14]. Several studies have shown that fluoride can induce changes in the physical structure and biochemistry of the brain, thereby affecting the intellectual development of children [15]. The membrane structure of neurons in brain tissue is rich in polyunsaturated fatty acids. The most vulnerable substrates of free radicals are polyunsaturated fatty acids, which cause lipid peroxidation [16]. Studies have shown that brain tissue suffers the most obvious harm by free radicals during fluorosis, and that fluorosis can cause an increase in OS. An increase in the latter can lead to apoptosis. Fluoride also affects cellular signaling pathways [17]. Silent information regulator (SIRT)1 is a histone deacetylase that can participate in OS, the inflammatory response, and neuroprotection via deacetylation [18]. Studies have shown that SIRT1 can activate downstream brain‐derived neurotrophic factor (BDNF). The latter is implicated in neural survival/development, and increased levels of SIRT1 can improve learning and memory dysfunction [19, 20]. BDNF can affect neuronal function through tyrosine kinase receptor B (TrkB)‐mediated activation of the downstream phosphatidylinositol 3‐kinase (PI3K)/protein kinase B (AKT) signaling pathway [21]. The latter is expressed widely in various tissues and organs of the body, participating in the proliferation, apoptosis, migration, and differentiation of cells, and is closely associated with nerve regeneration. In the PI3K/Akt pathway, activated Akt increases IKKα phosphorylation of mitogen‐activated protein kinase (MAPK) and nuclear factor‐kappa B (NF‐κB) pathways to regulate intracellular cascade reactions. The MAPK/NF‐κB signaling pathway aids regulation of the release of oxidative inflammatory factors. SIRT1, BDNF, and the MAPK/NF‐κB signaling pathway participate in regulation of the cascade response of neurons [22].

China has a wide range of areas in which fluorosis is prevalent. The prevention and control of fluorosis is a serious issue in China. Moreover, the lack of efficacious drugs for the treatment of fluorosis remains a problem that threatens the health of Chinese people. The perennial herb *Asparagus officinalis* (ASP) is rich in polysaccharides, saponins, flavonoids, trace elements, and other ingredients. ASP is being planted vigorously in China because it can be used in traditional Chinese medicine (TCM) formulations. Drug trials have shown ASP to have antioxidation, antiaging, and immune system‐enhancing effects [23]. TCM preparations have the characteristics of multiple components, multiple targets, and multiple effects, and carry an assortment of biological effects. Wang et al. found that ASP could promote urinary excretion of fluoride and had a strong scavenging effect for oxygen free radicals, and that ASP could improve the behavior of rats suffering from fluorosis [24].

**FIGURE 1:**
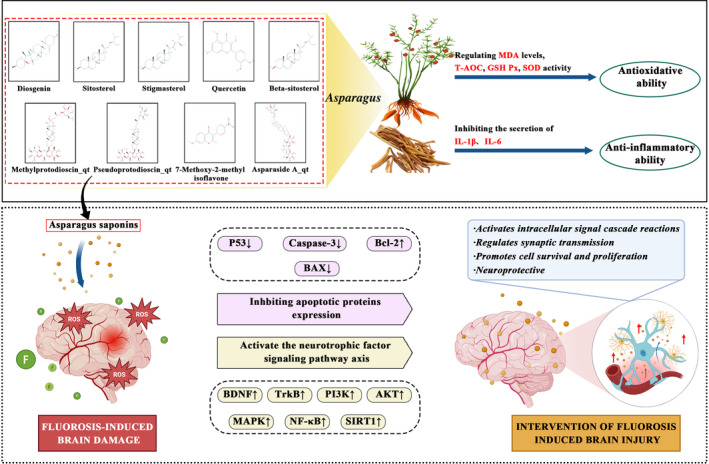
Network pharmacology and in vivo experimental verification of ASP for the prevention and treatment of the brain damage elicited by fluorosis.

Network pharmacology (NP) has emerged worldwide in recent years. It integrates technologies and content from multiple disciplines (e.g., systems biology, multidirectional pharmacology, computational biology, and network analysis) to construct a multiple‐level network. NP can be used to explore the correlation between a drug and disease, discover drug targets, and guide the development of new agents. Mechanistically, this strategy can associate drugs and diseases, predict and identify new drug targets effectively, and quantitatively represent the key links of the overarching regulatory actions of drugs (including crucial molecules, pathways, or modules) to explain the interaction between drugs and cells accurately [25]. How TCM formulations work has not been discovered because of their complex composition and the moieties involved in diseases. We analyzed the compounds present in ASP using NP. We explored the main targets of ASP in preventing and treating the brain damage caused by fluorosis, postulated a mechanism of action, and provided a foundation for application of the anti‐fluoride and anti‐oxidation effects of ASP. A flowchart of this study is shown as Figure 1.

## 2 Methods and Materials

### 2.1 Analysis of NP Targets

#### 2.1.1 Determination of the Active Ingredients of ASP

The Traditional Chinese Medicine Systems Pharmacology database (TCMSP; www.tcmspw.com/tcmsp.php/) provides the characteristics (absorption, distribution, metabolism, and excretion) of the chemical components from ASP in vivo [26]. The active components of ASP were screened for oral bioavailability (OB) ≥30%, and drug‐likeness (DL) ≥0.18. We used the databases GeneCards (www.genecards.org/), Online Mendelian Inheritance in Man (OMIM; www.omim.org/), and DisGeNET (www.disgenet.org/web/DisGeNET) to identify the genes associated with brain damage due to fluorosis. We used R 4.2.1 (https://cran.r‐project.org/) to identify the intersection of the targets of fluorosis‐induced brain injury and drug targets, which is a potential therapeutic target.

The Search Tool for the Retrieval of Interacting Genes/Proteins (STRING) database (https://string‐db.org/) has experimental validation and prediction functions for target‐to‐target interactions, obtaining protein–protein interaction (PPI) data. We employed Cytoscape 3.7.2 (www.cytoscape.org/) to construct a potential target PPI network. We used the “Network analyzer” function to assess the topological properties of the network, and thenobtained the efficacious ingredients for preventing and treating brain damage caused by fluorosis in ASP. The Gene Ontology (GO) (http://geneontology.org/) and Kyoto Encyclopedia of Genes and Genomes (KEGG) (www.genome.jp/) databases were employed for enrichment analyses of gene functions and signaling pathways, respectively, using R 4.2.1.

### 2.2 Experimental Validation

#### 2.2.1 Preparation of ASP

ASP was obtained as tablets via Guizhou Ren Ji Tang (Guiyang, China). ASP tablets (50 g) were weighed, followed by addition of 10‐times volume of water and weighing. Soaking was undertaken for 2 h. Extraction was undertaken twice by refluxing (1 h on each occasion). Extraction and filtration was carried out, and filtrates combined. The reduction in weight was compensated with water. Finally, we concentrated the solution at 70°C under reduced pressure to 1 g/mL of the raw drug, which was stored at −20°C.

#### 2.2.2 Construction of a Rat Model of Fluorosis

The protocol for animal experiments was approved (AHQU20210515A) by the Animal Ethics Committee of the First Affiliated Hospital of Guizhou University of Traditional Chinese Medicine (Guiyang, China).

Male specific pathogen‐free Sprague–Dawley rats (100–120 g) supplied by the Animal Experiment Center of Guizhou Medical University (SCXK [Qian] 2021‐0001) in Guiyang, China, were used. The ambient temperature of the room in which rats were housed was 20–25°C and humidity was 60% ± 20%. A 12‐h light–dark cycle was initiated. The exposure dose was set according to the human dose and half the lethal dose of soluble fluoride (Zhanwang Chemical Reagents, Wuxi, China). Adaptive feeding was done for 1 week. Rats were divided randomly into five groups of 20: control (drinking purified water), fluoride (drinking 100 mg/L of sodium fluoride water), low‐dose ASP (200 mg/kg of ASP+100 mg/L of sodium fluoride water), medium‐dose ASP (400 mg/kg of ASP+100 mg/L of sodium fluoride water), and high‐dose ASP (600 mg/kg of ASP+100 mg/L of sodium fluoride water). Rats in each ASP group were given ASP decoction by gavage 1–2 times (1‐mL each time) for 90 consecutive days. Their growth and development during these 90 days rats were documented. Blood from the femoral artery was sampled. Rats were killed after 90 days of exposure to fluoride and the induction of anesthesia. Brain tissue was stored at −80°C.

### 2.3 Test Indicators

#### 2.3.1 Dental Fluorosis

We recorded the degree of tooth damage in each group of rats each week. Before rats were killed, their teeth were photographed. The *Diagnostic Standard for Dental Fluorosis* (WS‐T208‐2011) suggests that DF can be divided into four grades: “normal” (surface is smooth and enamel is translucent), “mild” (some chalky and opaque areas on the tooth surface, with wear and tear), “moderate” (many chalky and opaque areas on the tooth surface, resulting in severe tooth wear), and “severe” (teeth have yellow‐brown plaques, loss of luster, linear or furrowed defects, and deep staining within dents).

#### 2.3.2 Bodyweight

After 90 days of administration and modeling, the bodyweight of rats was measured with an electronic scale (Huazhi Scientific Instruments, Beijing, China) and recorded.

#### 2.3.3 Urinary Fluoride Level

After fluoride exposure, the 24‐h urine of rats was obtained using a metabolic cage. The supernatant was centrifuged (1500 × *g*, 5 min, room temperature) for preservation. The fluoride‐ion content in urine was determined in accordance with WS/T 30‐1996 (“method for the determination of fluoride ion selective electrode”) using fluoride ion analyzer (Precision Scientific Instruments, Shanghai, China).

#### 2.3.4 Fluoride Concentration in Bone

After fluoride exposure and killing, the right femur was removed, followed by elimination of muscles and fat. The femur was dried in an oven at 105°C. After high‐temperature ashing, femoral fluoride content was measured in accordance with WS/T 30‐1996 using fluoride ion analyzer (Precision Scientific Instruments).

#### 2.3.5 Fluoride Concentration in Blood

Anesthesia was induced after the end of the feeding cycle. Blood was obtained from the femoral artery from each group of rats. Blood samples were centrifuged (1500 × *g*, 10 min, room temperature). Serum was stored at −80°C. The fluoride level in blood was measured in accordance with WS/T 30‐1996.

#### 2.3.6 Level of Oxidation Markers in Blood

Anesthesia was induced after the end of the feeding cycle. Blood was obtained from the femoral artery from each group of rats. Blood samples were centrifuged (1500 × *g*, 10 min, room temperature). Serum was stored at −80°C. The serum level of glutathione peroxidase (GSH‐Px) was measured using a method based on dithio‐dinitrobenzoic acid. We measured the activity of superoxide dismutase (SOD) using a method based on xanthine oxidase. The serum level of malonaldehyde (MDA) was measured using a method based on thiobarbituric acid. The total antioxidant capacity (T‐AOC) was determined by measuring the amount of Fe^3+^ reduction. The kits for these assays were sourced from Jiancheng Bioengineering Institute (Nanjing, China). A spectrophotometer (721 series; Jinghua Experimental Equipment, Shanghai, China) was employed to measure serum levels of MDA, T‐AOC, and GSH‐Px and SOD activity according to the instructions of reagent kits.

#### 2.3.7 Levels of Inflammatory Factors in the Brain

After fluoride exposure, the induction of anesthesia was undertaken, and rats were killed. Brain tissue was removed rapidly. Brain tissue (20 mg) was taken from each rat, followed by addition of 0.9% sodium chloride solution in a 1:1 ratio. After homogenization on ice, we undertook centrifugation (1500 × *g*, 10 min, room temperature) and extracted the supernatant. Levels of interleukin (IL)‐1β and IL‐6 were measured using enzyme‐linked immunosorbent assay (ELISA) kits from Jiancheng Bioengineering Institute.

#### 2.3.8 Pathologic Morphology of the Brain

After fluoride exposure, the induction of anesthesia was undertaken, and rats killed. Brain tissue was removed rapidly followed by fixation in paraformaldehyde solution for 2 days. Dehydration was undertaken using a graded series of ethanol solutions. We used transparent xylene and paraffin embedding to create tissue slices of thickness 5 μm. Next, we undertook dewaxing with xylene, and hydration using a graded series of ethanol solutions. Staining was done for 5–8 min using Harris hematoxylin solution, 1% hydrochloric acid ethanol differentiation, 0.6% ammonia water reverse blue, eosin staining solution staining for 1–3 min, gradient ethanol dehydration, xylene transparency, and neutral gum sealing. Slides were observed under a microscope (Leica, Wetzlar, Germany) at 200× magnification.

#### 2.3.9 Brain Ultrastructure

After fluoride exposure, the induction of anesthesia was undertaken, and rats were killed. Brain tissue was removed rapidly and placed on ice. Next, 3% glutaraldehyde was infused, followed by rapid fixation in 1% osmic acid at 4°C for 2–4 h. Dehydration was undertaken using a graded series of ethanol solutions, followed by embedding in Epon 812 overnight (Solarbio, Beijing, China). Tissue was cut into sections of thickness 50 nm using an ultra‐thin slicer. Then, sections were double‐stained with uranium acetate and lead citrate, followed by observation and under a transmission electron microscope (H‐600 series; Hitachi, Tokyo, Japan).

#### 2.3.10 Western Blotting

Sample (~100 mg) was transferred to an Eppendorf tube on ice. RIPA lysis buffer and a cocktail of protease inhibitors were added, followed by homogenization at low temperature and centrifugation (13 500 *g*, 15 min, 4°C). The supernatant was collected to measure the protein concentration using a bicinchoninic acid kit (Solarbio, Beijing, China). Western blotting was used to measure expression of SIRT1/BDNF/TrkB signaling pathway‐related proteins in brain tissue. Sodium dodecyl sulfate–polyacrylamide gel electrophoresis was used to isolate equal amounts of proteins (30 μg). Proteins were transferred to polyvinylidene difluoride membranes (PVDFMs; 0.45 μM; Millipore, Bedford, MA, USA). PVDFMs were sealed with 5% bovine serum albumin at room temperature for 1 h. The corresponding primary antibodies (SIRT1, BDNF, TrkB, B‐cell lymphoma (Bcl)‐2, caspase‐3, NF‐κB, PI3K, Akt, MAPK, and glyceraldehyde 3‐phosphate dehydrogenase) were incubated overnight at 4°C. PVDFMs were incubated with horseradish peroxidase‐labeled secondary antibodies for 1 h at 37°C. An electrochemiluminescence reagent (Boster Biotechnology, Beijing, China) was employed for color development. A gel‐imaging system was used to scan images. ImageJ (US National Institutes of Health, Bethesda, MD, USA) was employed to quantitatively analyze the grayscale value of protein bands according to grayscale readings normalized to the GAPDH level. PVDFMs were probed with primary antibodies (1:1000 dilution) from Beyotime Institute of Biotechnology (Shanghai, China).

### 2.4 Statistical Analyses

Statistical evaluations were carried out using Prism 8.3.1 (GraphPad, La Jolla, CA, USA). Data are the mean ± SD. Differences between two groups were compared using the Student's *t*‐test. Differences between multiple groups were analyzed using one‐way ANOVA. Further comparisons between two groups were made using the LSD test. *p* < 0.05 was considered significant.

## 3 Results

### 3.1 Active Ingredients of ASP

Search of the TCMSP database revealed seven active ingredients of ASP diosgenin, pseudoprotodioscin_qt, quercetin, beta‐sitosterol, sitosterol, stigmasterol, and 7‐methoxy‐2‐methyl isoflavone (Table 1) corresponding to 172 drug targets (Table S1).

**TABLE 1:** Active compounds found in ASP based on the TCMSP database.

Mol ID	Molecule name	Structure	OB (%)	DL
MOL000546	Diosgenin	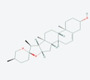	80.88	0.81
MOL003891	Pseudoprotodioscin_qt	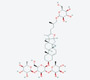	37.93	0.87
MOL000098	Quercetin	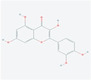	46.43	0.28
MOL000358	Beta‐sitosterol	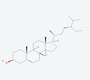	36.91	0.75
MOL000359	Sitosterol	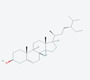	36.91	0.75
MOL000449	Stigmasterol	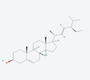	43.83	0.76
MOL003896	7‐Methoxy‐2‐methyl isoflavone	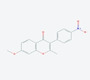	42.56	0.20

### 3.2 PPI Networks

**FIGURE 2:**
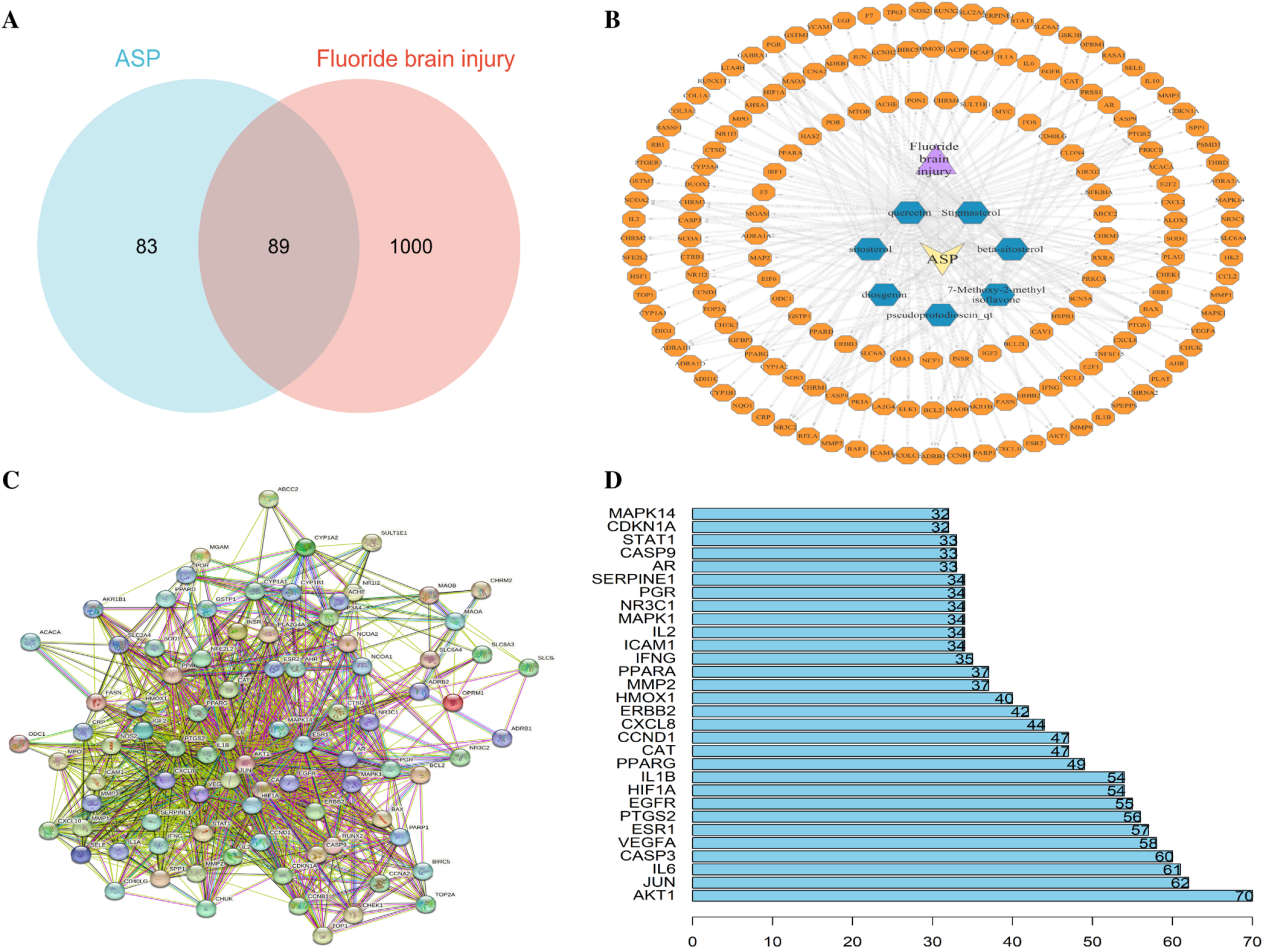
Drug–active ingredient–target network and Venn diagram. (A) Venn diagram. Pink denotes the brain damage caused by a fluorosis target, light‐blue denotes the ASP target, and the intersection region represents the ASP and brain damage caused by fluorosis‐crossover targets. (B) Drug–active ingredient–target network. (C) Protein–protein interactions identified by the STRING database. (D) Top‐20 core nodes according to their degree value.

A total of 1089 targets associated with brain damage due to fluorosis treatment were obtained from OMIM and GeneCard databases. After the creation of Venn diagrams, 89 potential overlapping genes were identified from the active targets of ASP drugs and targets of fluorosis‐induced brain injury (Figure 2A). We built PPI data of potential targets from the STRING database and imported it into Cytoscape for visualization (Figure 2B). Then, we inputted the results into Cytoscape to construct a target PPI network for the prevention and treatment of fluorosis‐induced brain damage by *Asparagus* (Figure 2C). We also identified the top‐20 core genes according to their degree value (Figure 2D).

### 3.3 Analyses of Pathway Enrichment

**FIGURE 3:**
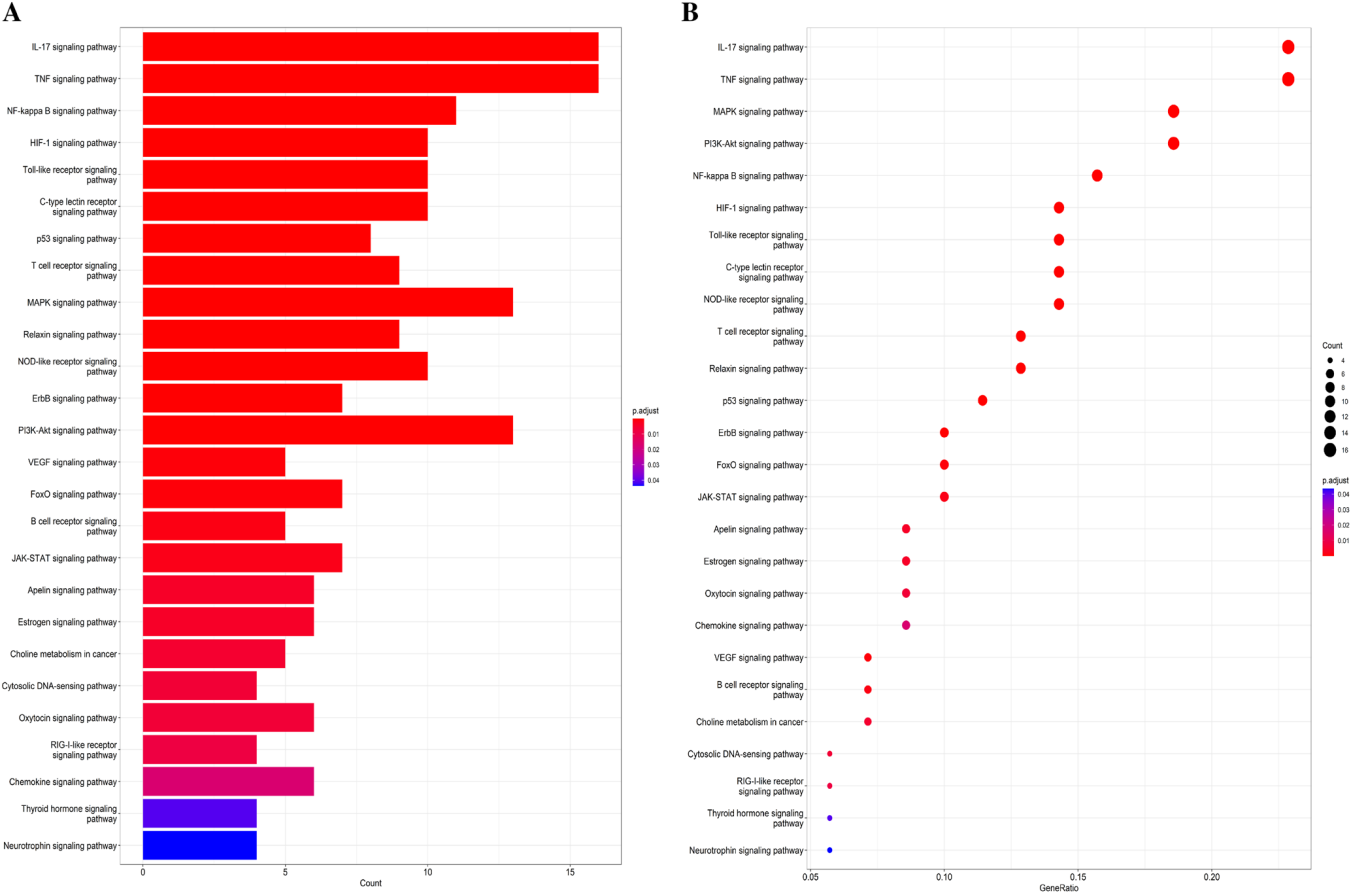
Exploration of the role of *Asparagus* in preventing and treating brain damage caused by fluorosis based on network pharmacology. (A) Enrichment analyses of key targets using the GO database. (B) Analysis of signaling‐pathway enrichment using the GO database.

We entered 89 targets of ASP for preventing and treating fluorosis‐induced brain injury into the Metascape database for analyses of enrichment of signaling pathways and function using the GO database and KEGG database, respectively. Functional‐enrichment analyses revealed “cytokine receptor binding,” “cytokine activity,” “antioxidant activity,” and “kinase activator activity” to be enriched. Values for the binding energy revealed the top‐20 biological processes (Figure 3A). Analyses of signaling‐pathway enrichment revealed 126 terms. The top‐26 signaling pathways were assessed for brain damage caused by fluorosis‐related signaling pathways according to *p* < 0.05 (Figure 3B). Of these, the most significant signaling pathways were “MAPK,” “PI3K‐AKT,” “NF‐κB,” “neurotrophin signaling pathway,” and other pathways related to brain damage caused by fluorosis. Among them, the neurotrophin signaling pathway was the most relevant to brain injury.

### 3.4 Establishment of a Rat Model of Fluorosis

**FIGURE 4:**
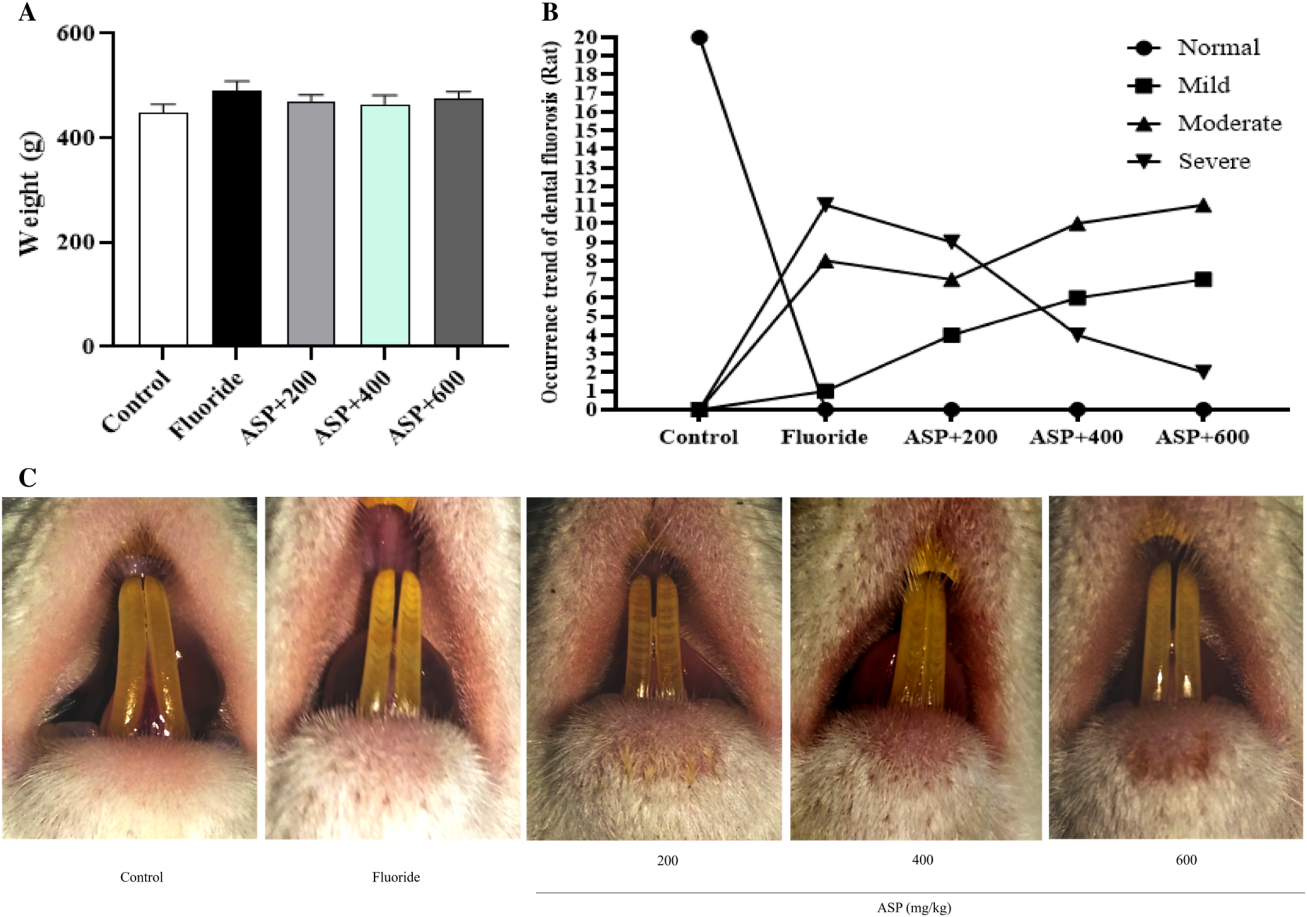
Creation of an animal model of fluorosis. (A) Bodyweight of rats in each group. (B) Number of rats with dental fluorosis in each group. (C) Severity of dental fluorosis of rats in each group.

We discovered the bodyweight of rats in the fluoride group to be greater than that in the control group and each ASP group. Also, the bodyweight of rats in each ASP group was greater than that in the control group, but these differences were not significant (*p* > 0.05) (Figure 4A). Rats in the control group did not experience DF. All rats exposed to fluoride had DF. This DF was primarily moderate and severe. Light, moderate, and severe DF accounted for one (5%), eight (40%), and 11 (55%) cases, respectively. The prevalence of DF of rats in each dose group of ASP was also 100%, but the proportion of rats with severe DF was reduced significantly: mainly light and moderate DF were observed. The proportion of light, medium, and severe DF of rats in the ASP group (200, 400, 600) was 20%, 35%, 45% (4, 7, 9), 30%, 50%, 20% (4, 6, 10), and 35%, 55%, 10% (2, 7, 11) (Figure 4B,C).

### 3.5 Fluoride Levels in Urine, Bone, and Blood

**FIGURE 5:**
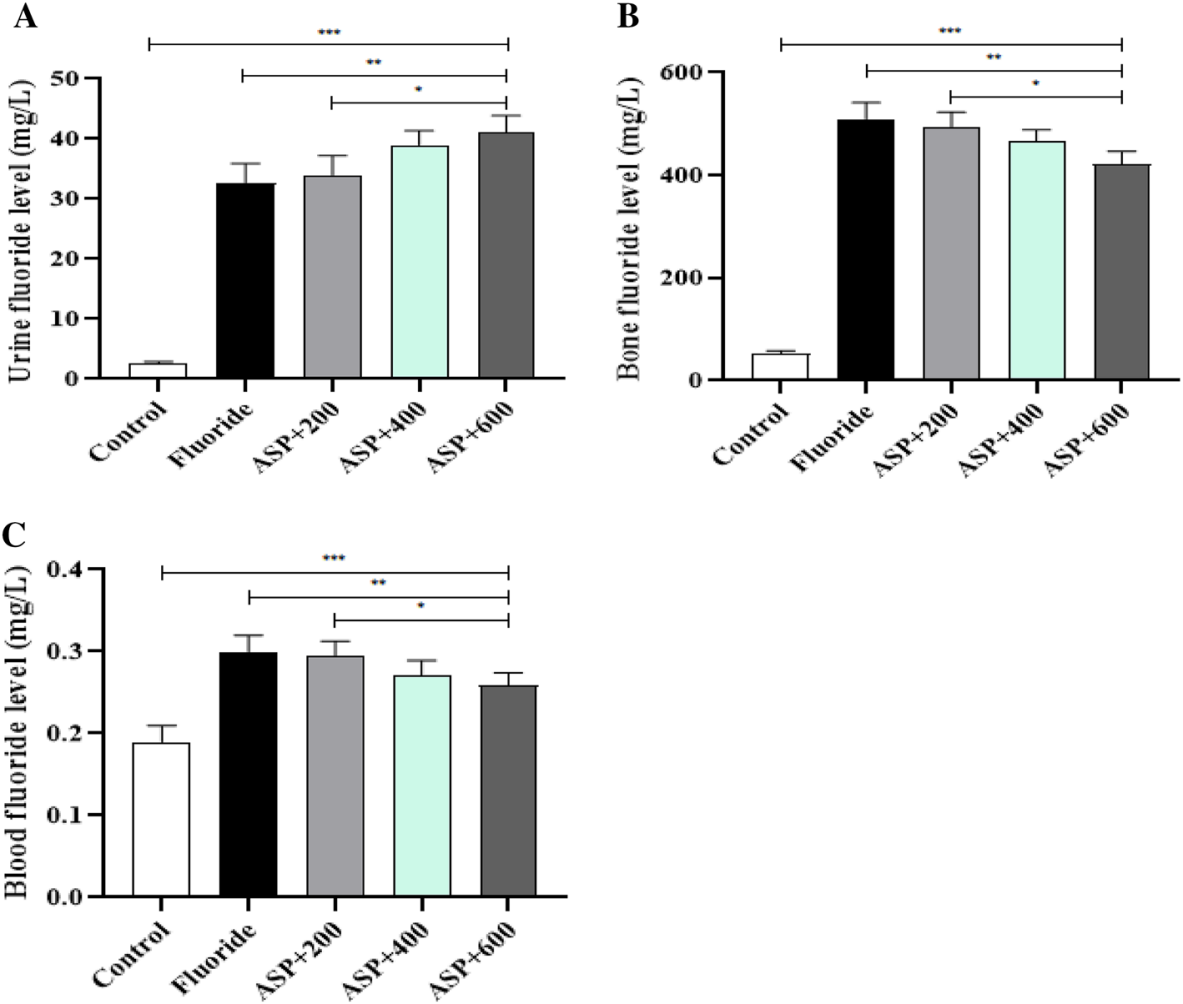
Effect of ASP on fluoride content in rats. (A) Urinary fluoride content in rats. (B) Fluoride content in rat bones. (C) Fluoride content in the blood of rats. ***p < 0.05 versus control group, **p < 0.05 versus fluoride group, and *p < 0.05 versus ASP.

Compared with the control group, fluoride levels in urine, bone, and blood in the fluoride group and each ASP group were significantly higher (*p* < 0.05). Compared with the fluoride group, with an increase in the ASP dose, the urinary fluoride level increased gradually, whereas the fluoride levels in bone and blood decreased gradually. We discovered a significant difference between the fluoride group and ASP‐medium‐dose group and ASP‐high‐dose group (*p* < 0.05). Compared with each ASP group, the urinary fluoride level increased gradually with an increase in the ASP dose, and the difference between the ASP‐medium‐dose group and ASP‐high‐dose group was significant (*p* < 0.05). The fluoride level in bone and blood decreased gradually (but significantly) between each group (*p* < 0.05) (Figure 5A–C).

### 3.6 Oxidation Factors in Serum

**FIGURE 6:**
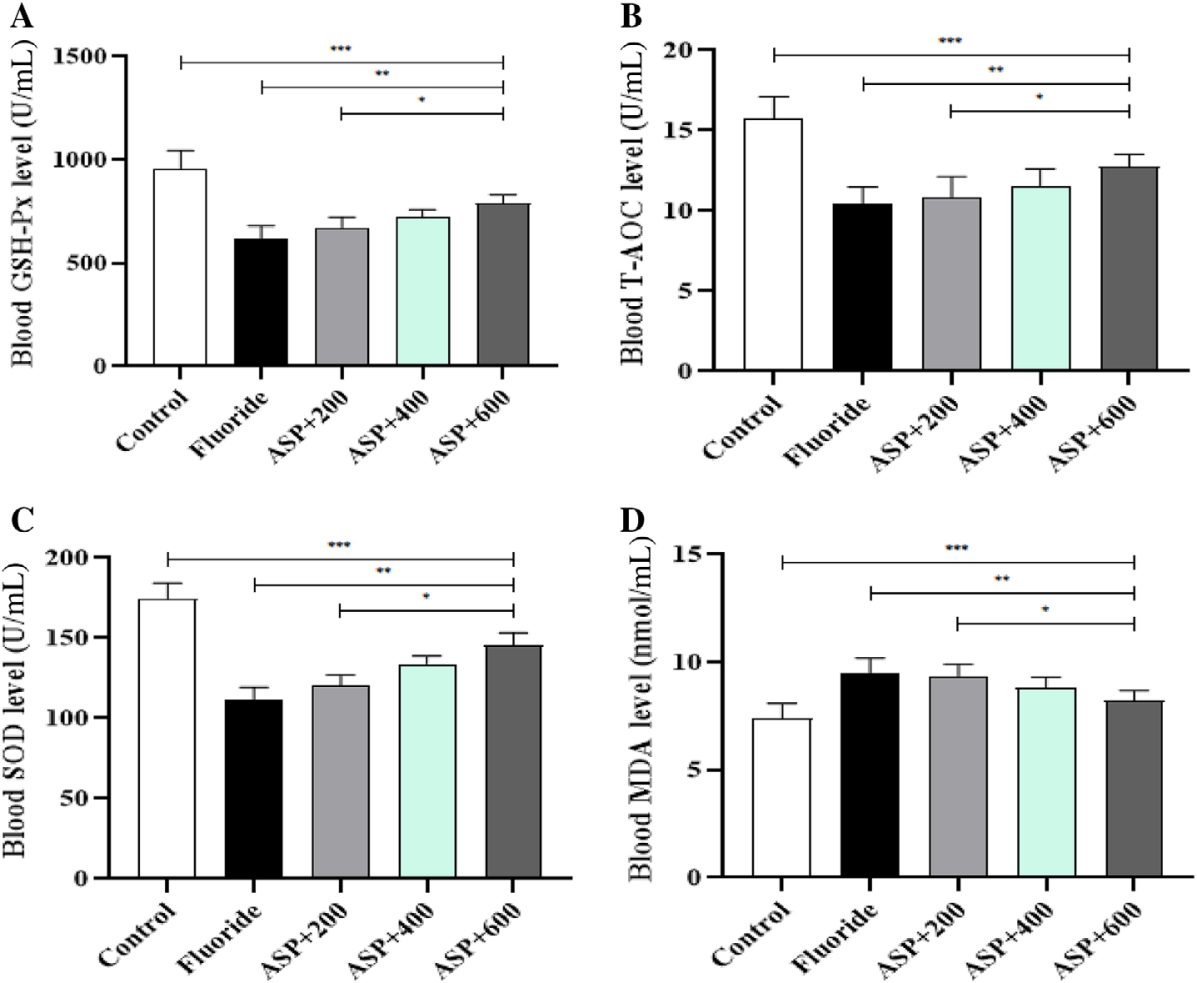
Effect of ASP on oxidation levels in the blood of rats. (A) Serum GSH‐Px level. (B) Serum T‐AOC level. (C) Serum SOD level. (D) Serum MDA level. ***p < 0.05 versus control group, **p < 0.05 versus fluoride group, and *p < 0.05 versus ASP.

Compared with the control group, levels of GSH‐Px and T‐AOC and SOD activity in the serum of rats in the fluoride group and each ASP group decreased significantly (*p* < 0.05). Compared with the fluoride group, levels of GSH‐Px and T‐AOC and SOD activity in each ASP group increased gradually, and the serum GSH‐Px level and SOD activity in rats in the fluoride group were significantly different from those of the three ASP groups (*p* < 0.05). There was a significant difference in the serum T‐AOC level for rats exposed to fluoride and that of the medium‐ and high‐dose groups of ASP (*p* < 0.05). Compared with each ASP group, levels of GSH‐Px and T‐AOC and SOD activity in the serum of rats increased gradually with an increase in the ASP dose. The difference in the GSH‐Px level and SOD activity between the three ASP groups was significant (*p* < 0.05), and there were significant differences in the T‐AOC level between the three ASP groups (*p* < 0.05). This effect was related to the effective dose (Figure 6A–C). The serum MDA content of rats in the fluoride group and each ASP group was significantly greater than that in the control group (*p* < 0.05). The serum MDA content of rats in the ASP groups was lower than that in the fluoride group, and the difference was significant compared with that in medium‐ and high‐dose groups (*p* < 0.05). Compared with each ASP group, the serum MDA content of rats fell gradually with an increment in the ASP dose, and the difference between groups was significant (*p* < 0.05) (Figure 6D).

### 3.7 Levels of Inflammatory Factors in Brain Tissue

**FIGURE 7:**
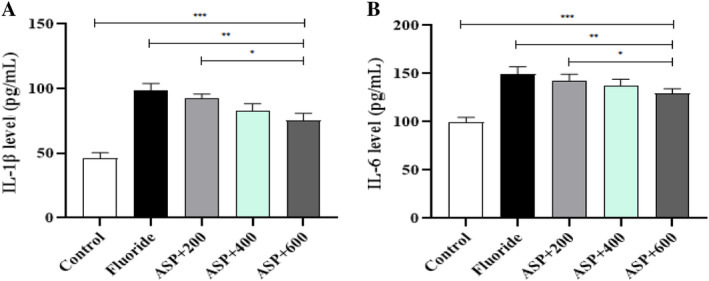
Effect of ASP upon inflammatory factors in the brain tissue of rats suffering from fluorosis. (A) IL‐1β concentration in brain tissue. (B) IL‐6 concentration in brain tissue. ***p < 0.05 versus control group, **p < 0.05 versus fluoride group, *p < 0.05 versus ASP.

Levels of IL‐1β and IL‐6 in brain tissue of rats in the fluoride group and each ASP group were significantly higher than those in the control group (*p* < 0.05). Levels of IL‐1β and IL‐6 in the brain tissue of rats in ASP groups were lower than those in the fluoride group, and the difference was significant compared with that in medium‐ and high‐dose groups (*p* < 0.05). Compared with each ASP group, levels of IL‐1β and IL‐6 in the brain tissue of rats decreased gradually with an increase in the ASP dose, and the difference between groups was significant (*p* < 0.05) (Figure 7).

### 3.8 Structure of Brain Tissue

**FIGURE 8:**
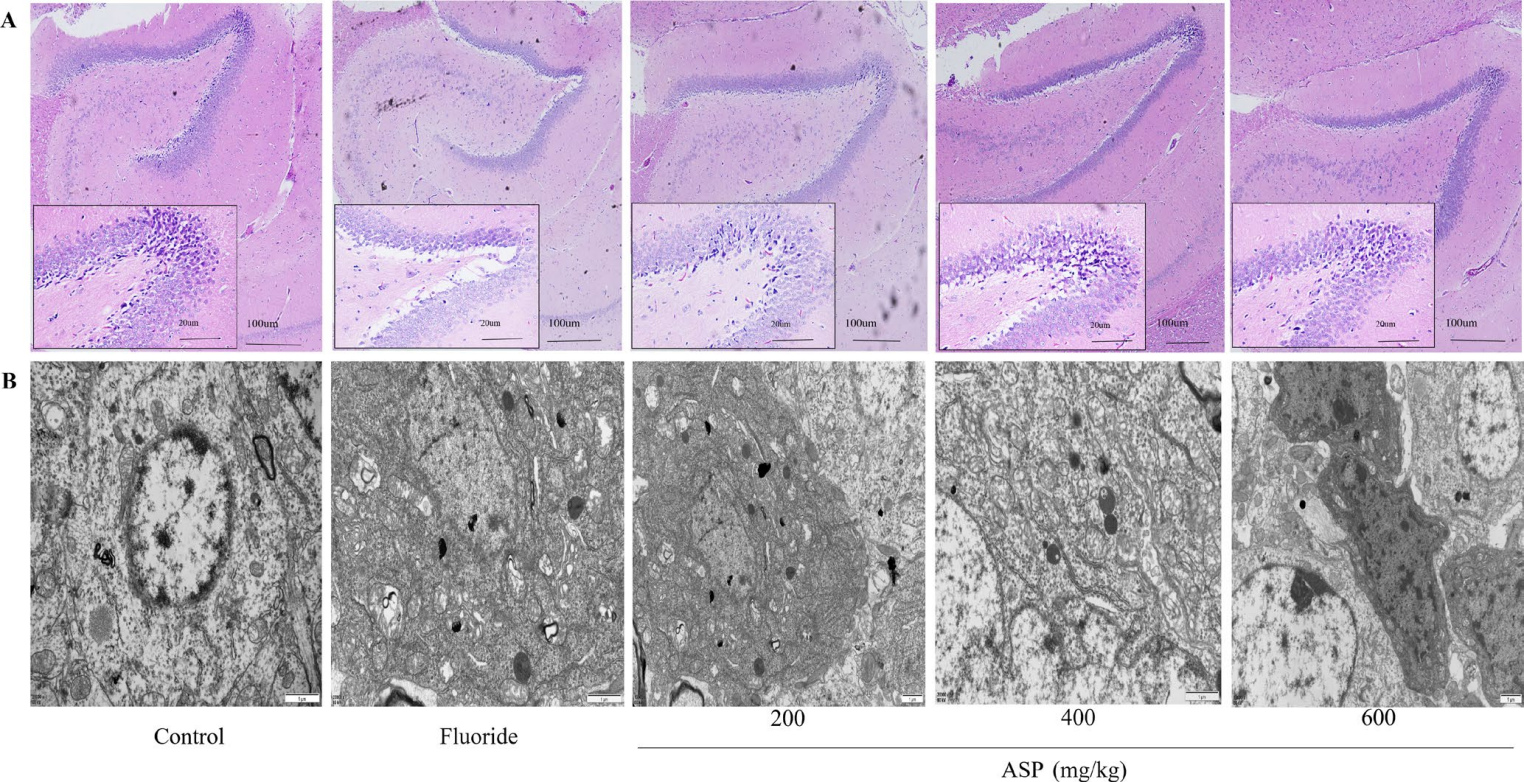
Effect of ASP on brain histopathology in rats suffering from fluorosis. (A) H&E staining of brain tissue showing pathologic morphology. (B) Ultrastructure of brain tissue.

H&E staining revealed that the hippocampal neurons of rats in the control group were arranged in a regular and orderly fashion, the cell structure was clear and complete, and there was no edema between cells. The neurons in the fluoride group were arranged sparsely, with irregular cell arrangement, intercellular swelling and infiltration, reduced number of astrocytes, cell swelling, and disappearance of nucleoli. Compared with each ASP group, with an increase in the ASP dose, the arrangement of hippocampal neurons of rats became gradually dense and ordered, the number of astrocytes increased gradually, the nucleolus became clear, and damage to brain tissue cells was relieved, which was improved significantly compared with that observed in the fluoride group (Figure 8A).

Transmission electron microscopy showed that neuron nuclei in the control group had a regular shape, the double‐layer structure of the cellular nuclear membrane was clear, chromatin in the nucleus was distributed evenly, and a multitude of organelles were distributed normally. In the fluoride group, the neuron membrane was folded, mitochondria were deformed, cristae had disappeared, vacuoles had formed, and chromatin was pyknotic and hyperchromatic. Compared with each ASP group, with an increase in the ASP dose, the morphology of neuron nuclei became regular gradually, the structure of the cellular nuclear membrane became clear gradually, chromatin aggregation was reduced, and many organelles were present (Figure 8B).

### 3.9 Expression of the Neurotrophin Signaling Pathway

**FIGURE 9:**
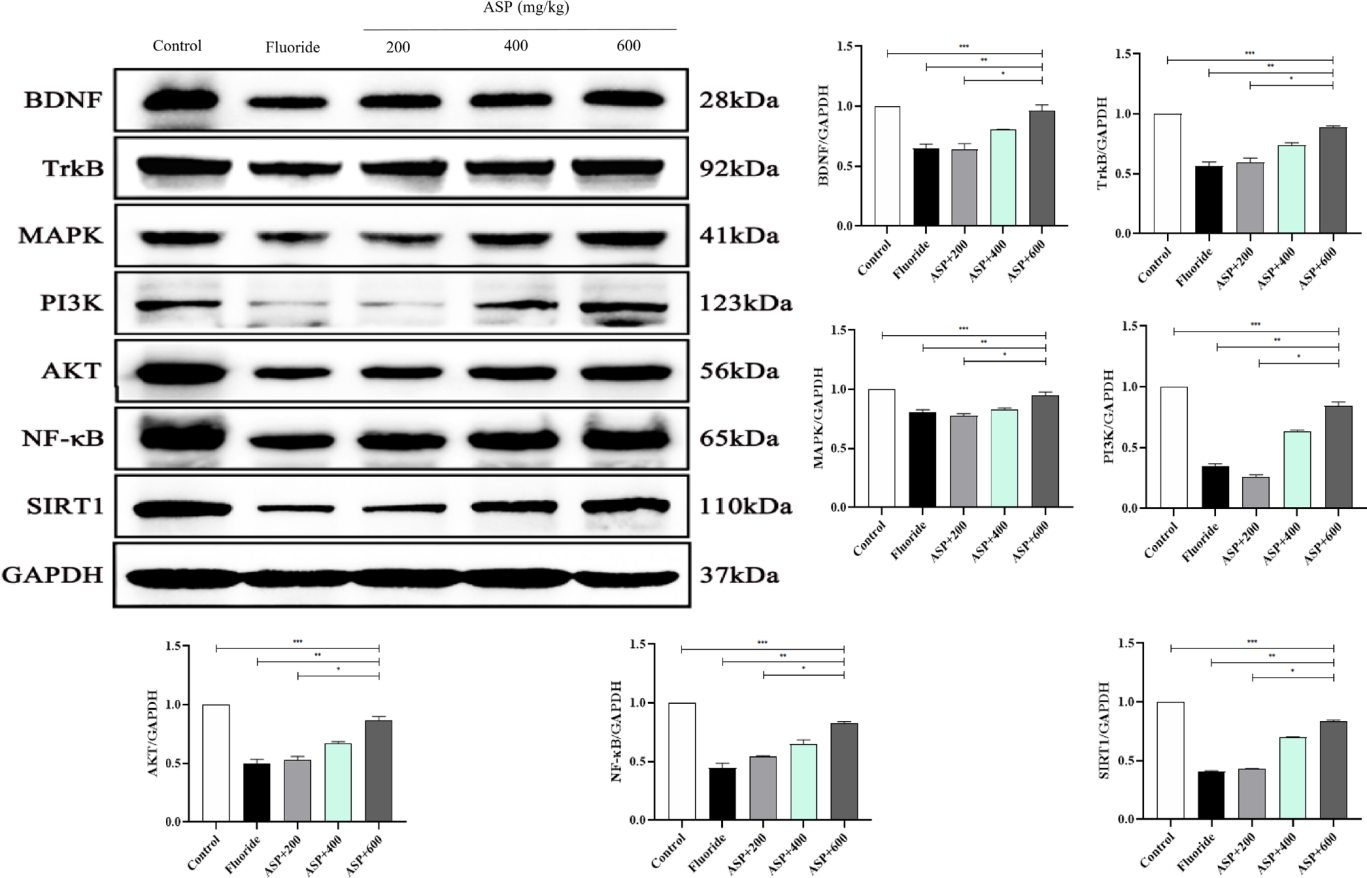
ASP mediates the SIRT1/BDNF/TrkB signaling pathway in protein expression in the brain tissue of rats suffering from fluorosis. ***p < 0.05 versus control group, **p < 0.05 versus fluoride group, and *p < 0.05 versus ASP.

**FIGURE 10:**
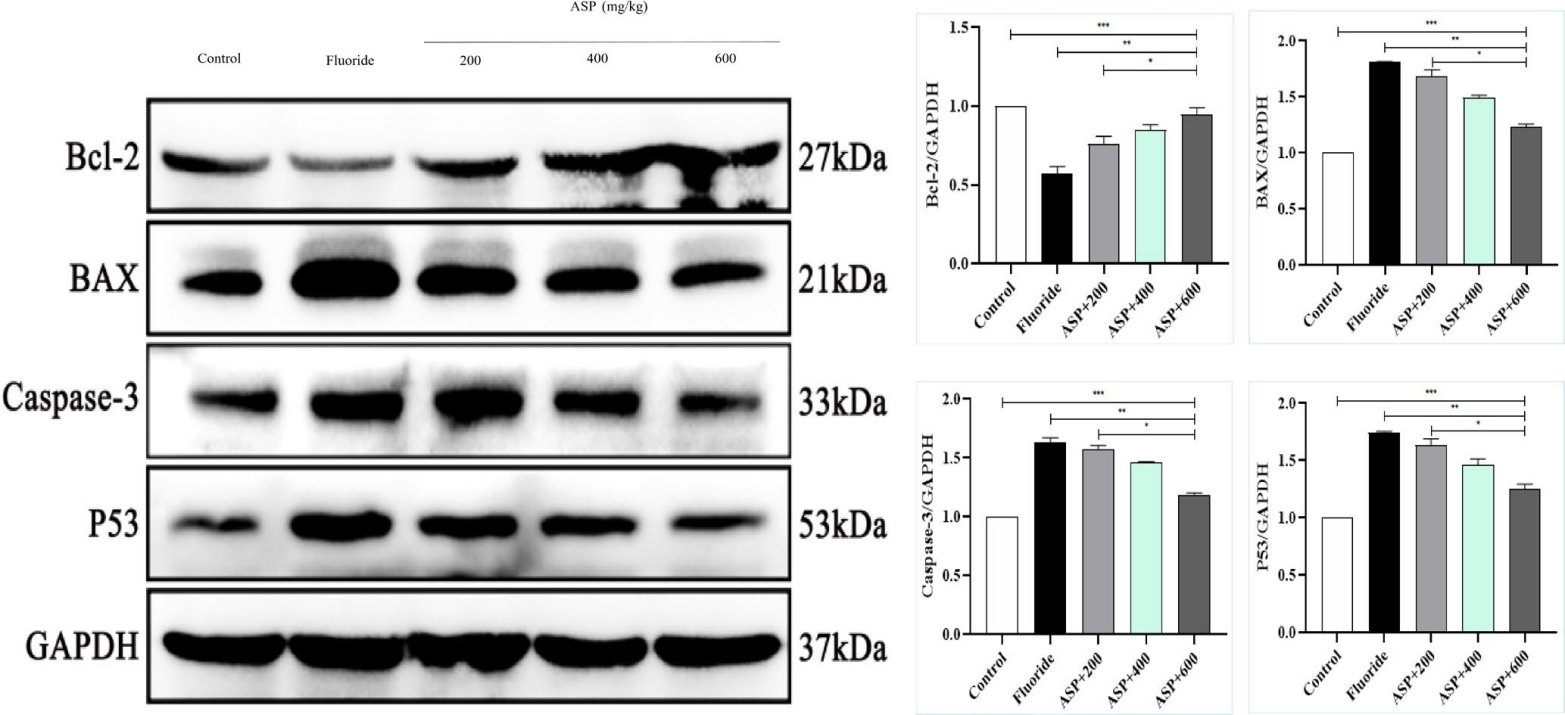
Expression of apoptotic proteins in the brain tissue of rats suffering from fluorosis treated with ASP. ***p < 0.05 versus control group, **p < 0.05 versus fluoride group, and *p < 0.05 versus ASP.

We discovered that the neurotrophin signaling pathway was a key target of ASP, and that the signaling pathways involved in brain injury were the most enriched. Therefore, we tested whether the BDNF/TrkB signaling pathway was implicated in the protective effect of ASP against brain injury. We analyzed the effect of ASP on the BDNF/TrkB pathway in brain tissue in rats suffering from fluorosis. Western blotting revealed that protein expression of BDNF, TrkB, SIRT1, MAPK, NF‐κB, PI3K, Akt, and Bcl‐2 in the brain tissue of rats exposed to fluoride was reduced significantly (Figure 9), and that expression of apoptotic proteins (Bax, caspase‐3, and p53) was increased significantly (Figure 10). With an increase in the ASP dose, expression of BDNF, TrkB, SIRT1, MAPK, NF‐κB, PI3K, Akt, and Bcl‐2 in brain tissue increased gradually, whereas that of Bax, caspase‐3, and p53 decreased gradually. These results showed that ASP participated in protection of the brain damage caused by fluorosis through the SIRT1/BDNF/TrkB signaling pathway.

## 4 Discussion

The neurotoxicity induced by oxidation products is considered to be the mechanism by which brain tissue is damaged by fluorosis. Peroxides penetrate the blood–brain barrier directly to pass into brain cells and accumulate in brain tissues, thereby damaging the nervous system in the brain [27]. Fluorosis causes peroxide accumulation in the brain, resulting in damage to and the apoptosis of neurons [28, 29]. Studies have shown that an increase in lipid peroxidation and a reduction in the activity of antioxidant enzymes in the brain tissue of rats with fluorosis leads to histopathologic changes, especially the swelling of mitochondrial neurons and expansion of endoplasmic reticuli [30, 31]. Studies have demonstrated that antioxidants can prevent the brain damage caused by fluoride [32]. However, the toxicity of artificial antioxidants can lead to DNA damage and the risk of malignancy [33]. Therefore, the safety of artificial antioxidants is controversial [34]. Antioxidant ingredients extracted from natural plants, as substitutes for artificial antioxidants, have attracted considerable attention [35]. ASP is used as a herbal medicine in China. ASP has powerful antioxidant, antiaging, antitumor, and immune system‐enhancing features [36]. Kamat et al. studied liver injury and found that an ASP extract had a significant effect in inhibiting protein peroxidation and a potential antioxidant effect [37]. Parihar and Hemnani studied damage to hippocampal neurons and found that an ASP extract could increase GSH‐Px activity and the concentration of reduced‐GSH, and reduce lipid peroxidation and protein carbonylation [38].

Research on the pharmacology of a TCM formulation focuses on extracting its components and studying the linear pattern of “single drug, single component, and single disease target” with a single pharmacological effect [39]. Treatment of a single disease with a single drug is difficult to apply because diseases are complex and under the influence of multiple factors. Hence, a “multicomponent, multi‐target” drug design has been recognized gradually. NP utilizes systems‐biology theory to analyze the “drug target disease” as a whole, which has many similarities with the overall theory of TCM. Therefore, NP has become an important discipline in exploring TCM mechanisms and has become popular. We used NP to screen efficacious targets and validated the mechanism of action of ASP in fluorosis‐induced brain injury by employing in vivo experiments.

Upon separation and purification of a crude extract of ASP, abundant chemical components in ASP (e.g., saponins, polysaccharides) were found to have significant pharmacologic activities [40, 41]. Several investigations have revealed that excessive fluoride intake can lead to DF and skeletal fluorosis, which are the most common markers of fluorosis [42]. A change in the urinary fluoride level is another specific marker of fluorosis [43]. We showed that, after intragastric administration of ASP in rats suffering from fluorosis, fluoride levels in bone and blood, and the degree of damage to teeth and bone tissue in rats, were significantly lower than those in the fluoride group, and that the urinary fluoride level was significantly greater than that in the fluoride group. These results suggest that ASP may improve bone damage by promoting the renal excretion of urinary fluoride, reducing fluoride levels in blood and bone, and reducing fluoride deposition in bone.

In health, the oxidative and antioxidant systems of the body are in a dynamic balance. If this balance is disrupted, then the body is prone to OS, which, ultimately, leads to damage and various diseases [44]. SOD, T‐AOC, and GSH‐Px are important components of the antioxidant system of the body. T‐AOC reflects the total antioxidant capacity of the body. SOD and GSH‐Px can reduce the accumulation of superoxide free radicals and peroxides in the body. Fluoride poisoning causes endogenous and exogenous oxidative‐stress damage, which is an important public‐health issue for the prevention and treatment of fluorosis [45]. Brain tissue is exceptionally dependent upon oxygen and abundant in polyunsaturated fatty acids. The strong oxidizing nature of fluoride leads to high levels of lipid peroxidation in brain tissue, leading to OS‐related damage [46]. Research has found that long‐term exposure to fluorosis significantly increases the OS level in rats, leading to the apoptosis of brain‐tissue cells. It has also been observed that a reduction in learning and memory ability is closely related to peroxide levels [46]. Experiments have revealed that brain tissue suffers the most obvious peroxide‐based damage after the body ingests a large amount of fluoride. Research has shown that ASP can reduce lipid peroxidation in organisms [47]. Our in vivo studies showed that levels of GSH‐Px and T‐AOC and SOD activity in the serum of rats in the fluoride group were significantly lower than those in the ASP group, the serum MDA level in the ASP group was significantly lower than that in the fluoride group, and that the effect was related to the ASP dose. Research has shown that fluorosis reduces the antioxidant capacity of the body significantly and leads to the accumulation of lipid peroxide, which is consistent with the theory that fluorosis causes damage due to free radicals [48]. OS leads to the release of some signaling molecules that activate the inflammatory response, which leads to changes in the internal and external *milieu* of cells and increases the accumulation of oxidative products. The mechanism of tissue damage elicited by oxidative inflammatory products is mutually causal [49, 50, 51]. The body is exposed to OS and an inflammatory microenvironment for a long time, and oxidative products and inflammatory factors accelerate the process of brain damage [52]. Construction of a rat model of fluorosis revealed a significant increment in levels of inflammatory factors in the brain tissue of rats with fluorosis. These research results suggest a correlation between OS, inflammation, and brain damage caused by excessive fluoride intake. The concentration of inflammatory factors in the ASP group was significantly lower than that in the fluoride group, and this effect was related to the ASP dose.

Epidemiological reports have shown that fluoride can accumulate in the central nervous system to cause brain‐tissue damage [7]. Studies have demonstrated that fluorosis can cause structural changes to neurons and brain functions, such as nuclear atrophy, mitochondrial swelling, and neurodegeneration [53, 54]. Those findings indicate a direct link between excessive exposure to fluoride and brain damage, but little is known about the mechanism behind these phenomena. BDNF and SIRT1 have neuroprotective effects [55]. SIRT1 is a deacetylase that participates in the regulation of apoptosis under stress, can enhance cellular activity and the ability of self‐repair and survival, reduce inflammatory reactions, and has a protective role in neuron injury [56]. SIRT1 can improve synaptic plasticity and protect nerves as well as learning and memory abilities by promoting the expression of neurotrophic factors (e.g., BDNF), antioxidant stress, and inhibiting neuroinflammation [57]. BDNF is the main neurotrophic factor in the brain that regulates synaptic plasticity and important cellular events, and is the foundation for neuronal activity, learning, and memory formation [58]. BDNF can regulate the release of brain neuroenzymes through the TrkB receptor, thereby affecting brain neuroregulation and behavioral changes [59]. Upon binding to TrkB, BDNF induces the dimerization and self‐phosphorylation of receptors, resulting in the localization of TrkB receptors in vivo and triggering an intracellular signal cascade, including several pathways (PI3K, NF‐κB, and MAPK) [60, 61]. The PI3K pathway activates Akt and inhibits Bad to promote cell survival [21]. Akt phosphorylation at a normal site also leads to inhibition of expression of pro‐apoptotic proteins, and upregulated expression of Bcl‐2 is related to reducing the risk of cell death [62]. The MAPK pathway regulates the growth and differentiation of cells. A coalition of BDNF and TrkB activates various intracellular signal‐cascade reactions, then regulates synaptic transmission, promotes the survival and proliferation of cells, and can be neuroprotective [63].

We identified, through use of NP, the potential drug targets of ASP for the prevention and treatment of the brain damage caused by fluorosis. We identified the core targets through analyses of PPI networks. The most important signaling pathways were MAPK, NF‐κB, PI3K‐Akt, neurotrophin, and other pathways related to brain damage caused by fluorosis. Among them, the neurotrophin signaling pathway is most relevant to brain injury. We speculate that ASP may prevent and protect against the brain damage caused by fluorosis through a SIRT1‐mediated BDNF/TrkB signaling pathway. We demonstrated that protein expression of BDNF, TrkB, MAPK, NF‐κB, PI3K, Akt, SIRT1, and Bcl‐2 in the brain tissue of rats exposed to fluoride was decreased significantly, and that expression of apoptotic proteins (Bax, caspase‐3, and p53) was increased significantly. We demonstrated that the brain damage elicited by fluorosis was associated with the regulation of multiple signaling pathways and overactivation of apoptosis. Our results suggest that ASP could improve the alterations in protein expression in rat brain tissue, reduce expression of apoptotic proteins and, ultimately, achieve an anti‐fluorosis effect. These results revealed ASP can participate in the protection of brain damage elicited by fluorosis through the SIRT1/BDNF/TrkB signaling pathway.

## 5 Conclusions

The pharmacologic mechanism of action of ASP on the prevention and treatment of brain damage caused by fluorosis was investigated through a combination of NP and in vivo experiments. We speculate that the anti‐fluorosis mechanism of action of ASP may be through promotion of renal fluoride excretion, reducing the levels of fluoride and inflammatory factors in the body, increasing the antioxidant level in the body, mediating the SIRT1/BDNF/TrkB signaling pathway to regulate the brain damage caused by fluorosis, and reducing the apoptosis of neurons. Our data provide a theoretical and experimental foundation for the use of TCM formulations against fluorosis.

## Author Contributions

F.W., Y.J.L., Y.L., and X.Z. conceived and designed the study; they had full access to all the data in the study and take responsibility for the integrity of the data and the accuracy of the data analyses. F.W., D.T., and Y.J.L. wrote the manuscript. D.M., Z.H., and Y.L. revised the manuscript critically. J.Z., X.Y., C.Z., and B.Y. undertook statistical analyses. All authors approved the final version of this manuscript.

## Conflicts of Interest

The authors declare no conflicts of interest.

## Supporting information


Figure S1.

Figure S2.

Table S1.

## Data Availability

The data that support the findings of this study are available from the corresponding author upon reasonable request.
