# Supplementary material for: Combining Network Pharmacology and Experimental Verification to Ascertain the Mechanism of Action of Asparagus officinalis Against the Brain Damage Caused by Fluorosis
Source: Environ Toxicol. 2024 Jul 23;40(4):509–23. doi: 10.1002/tox.24382 (PMC11911904; doi:10.1002/tox.24382)
Supplement: Supplementary file 1 — Figure S1. [file TOX-40-509-s003.pdf]

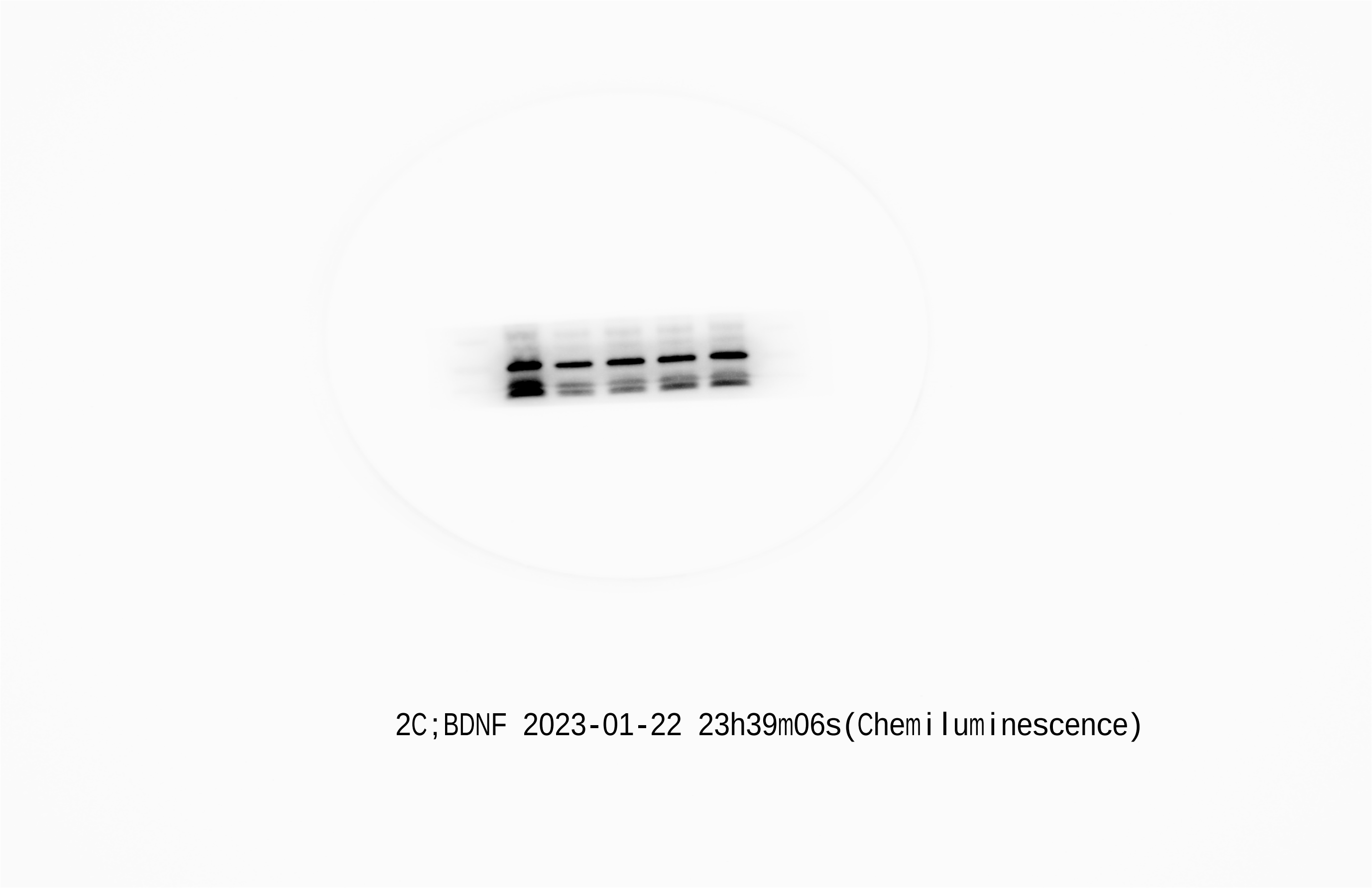
2C;BDNF 2023-01-22 23h39m06s(Chemiluminescence)

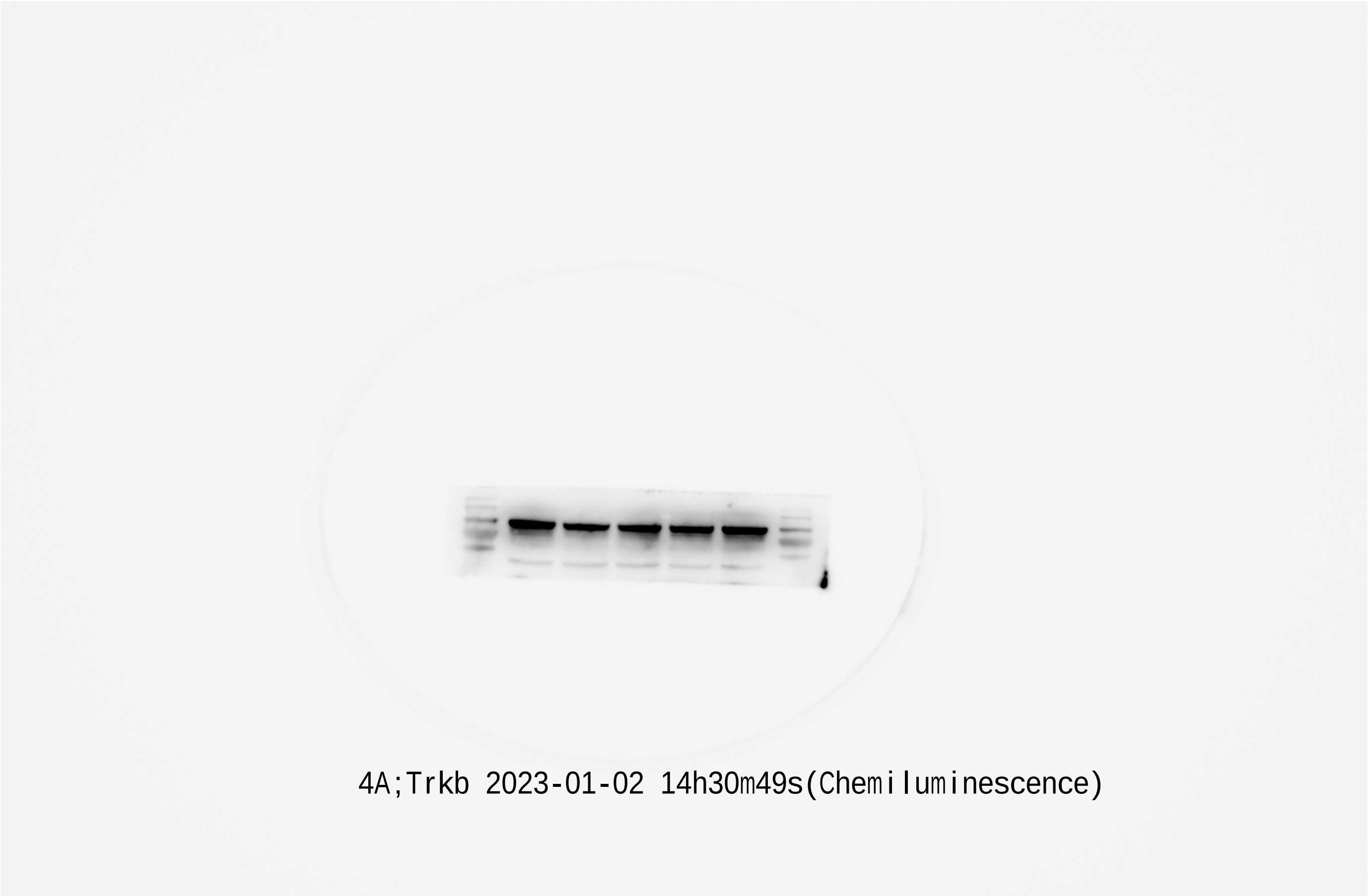
4A;Trkb 2023-01-02 14h30m49s(Chemiluminescence)

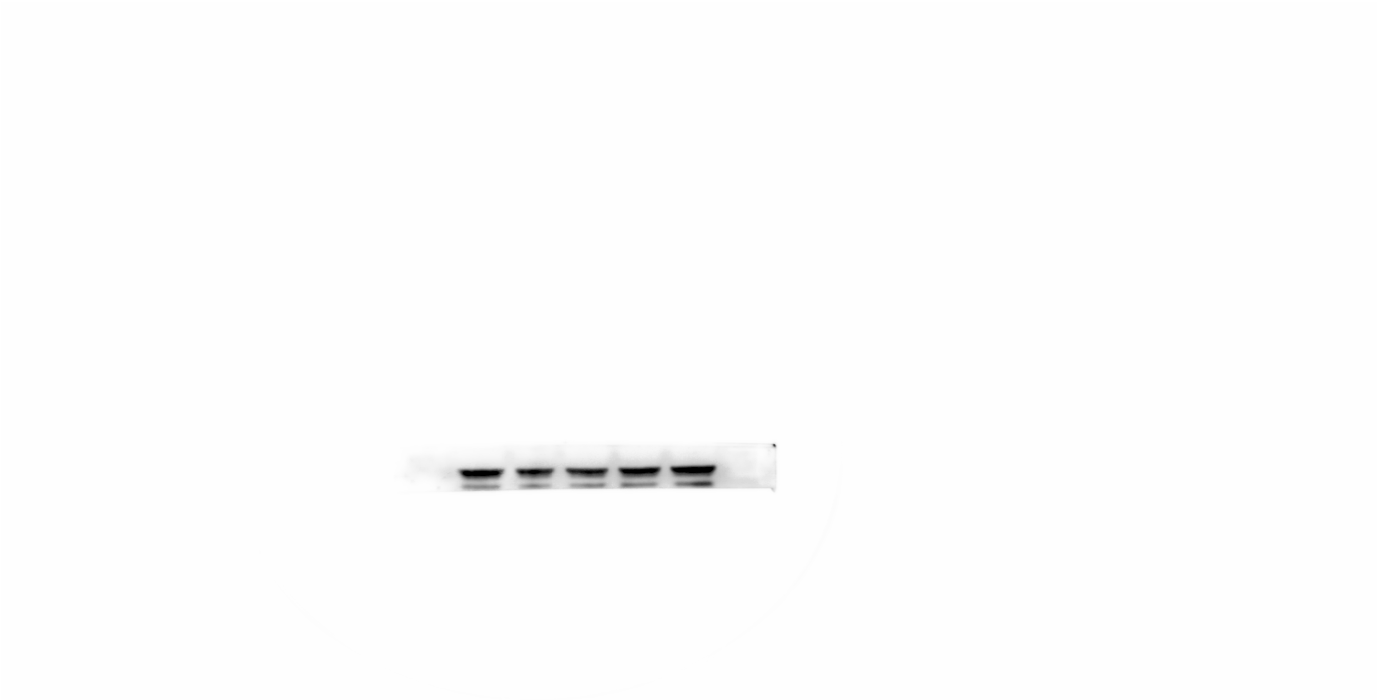

2C;MAPK 2022-12-29 16h21m14s(Chemiluminescence)

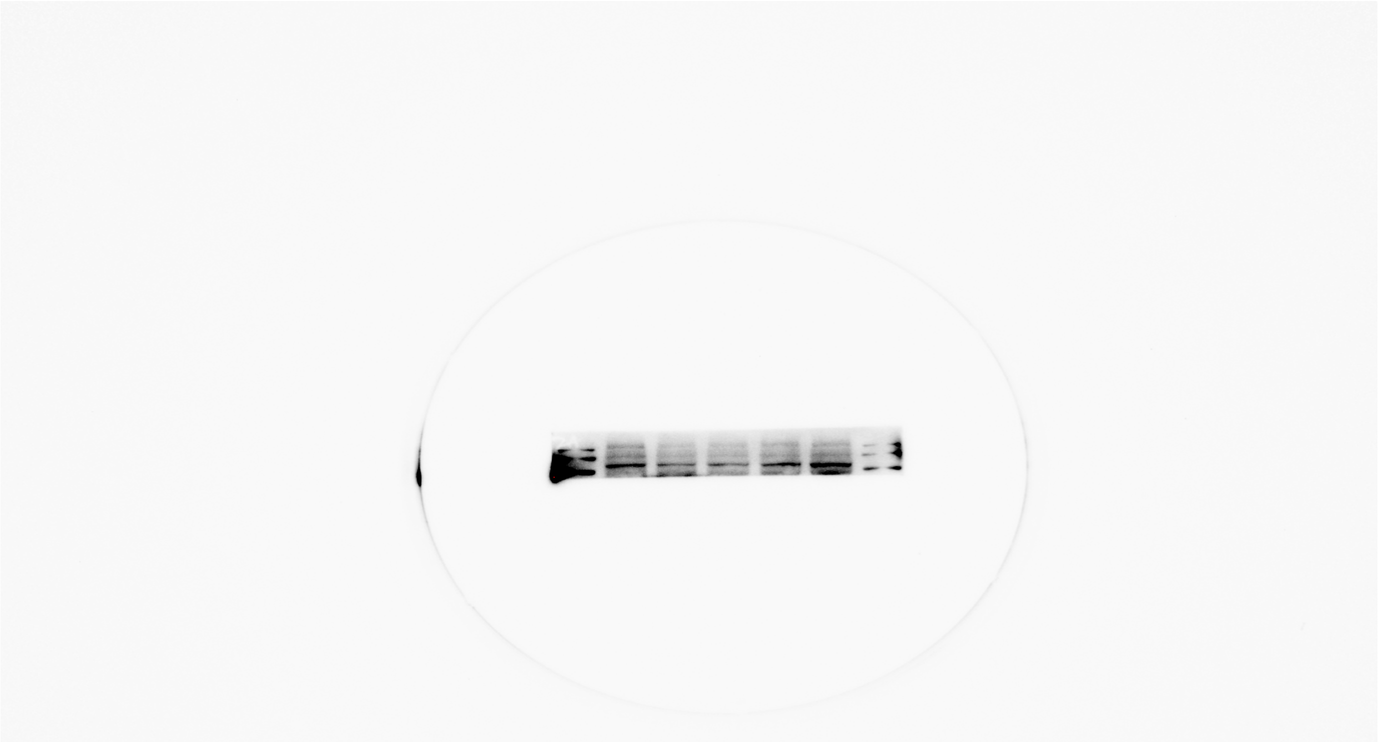

2A;PI3K 2022-12-28 14h04m34s(Chemiluminescence)

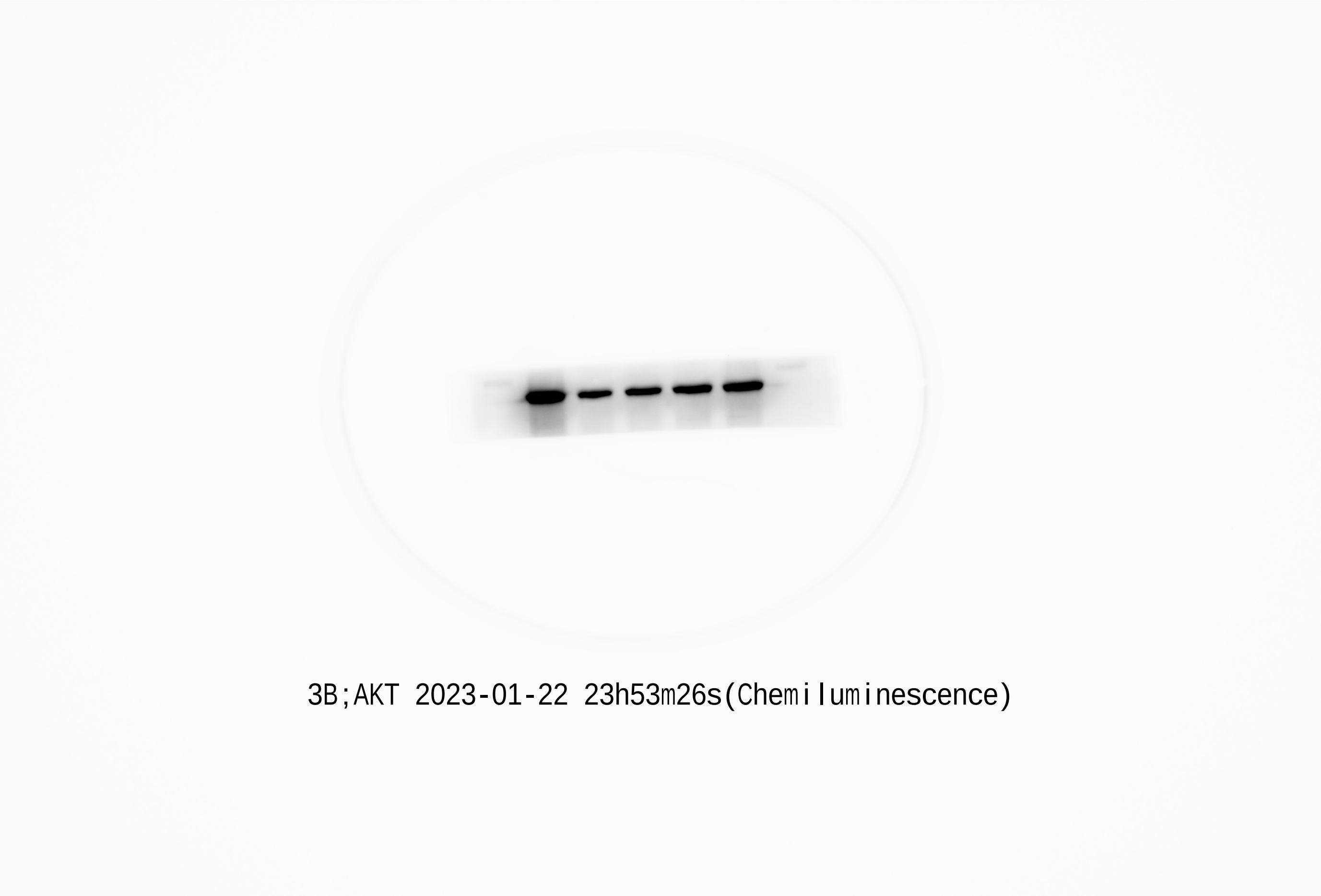
3B;AKT 2023-01-22 23h53m26s(Chemiluminescence)

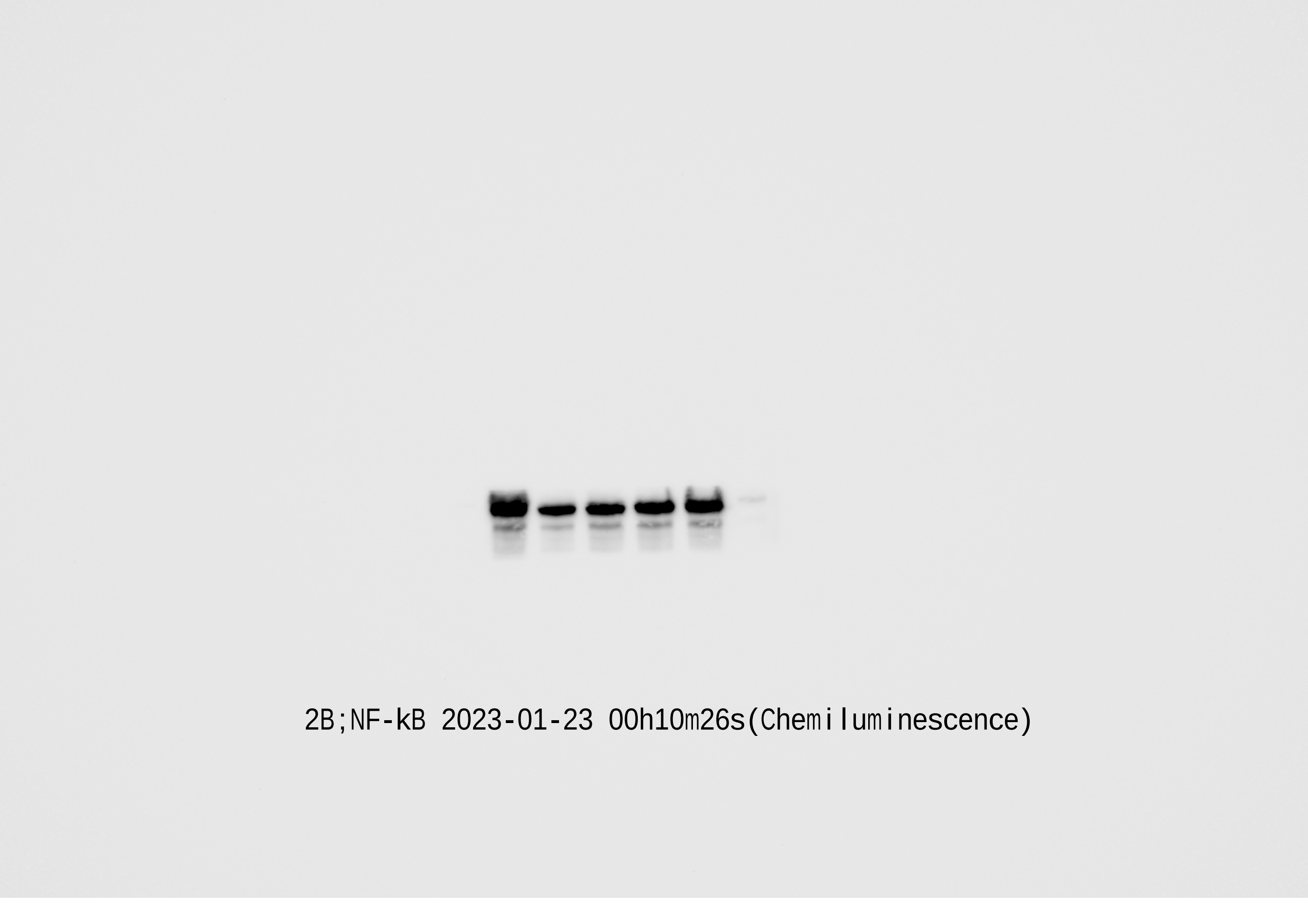
2B;NF-kB 2023-01-23 00h10m26s(Chemiluminescence)

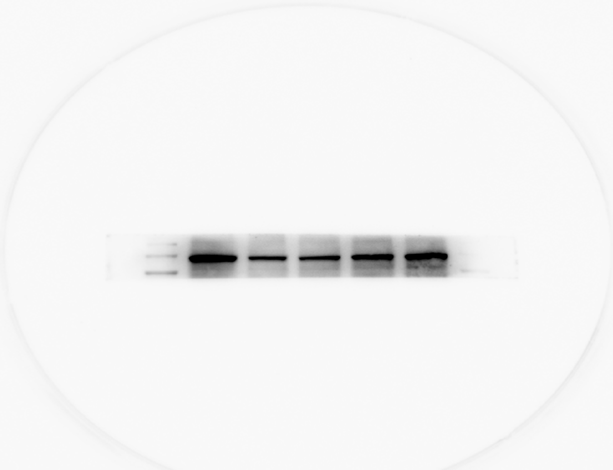

1A;SIRT1 2022-12-29 18h03m41s(Chemiluminescence)

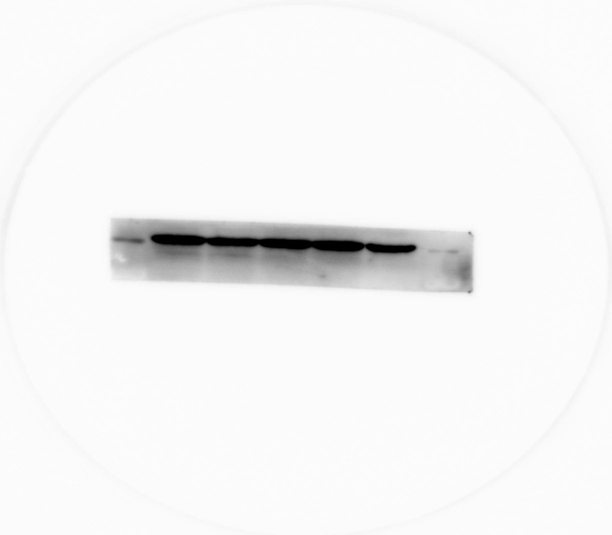

1C;GA 2022-12-27 13h20m16s(Chemiluminescence)

Figures S1
