# Supplementary material for: Combining Network Pharmacology and Experimental Verification to Ascertain the Mechanism of Action of Asparagus officinalis Against the Brain Damage Caused by Fluorosis
Source: Environ Toxicol. 2024 Jul 23;40(4):509–23. doi: 10.1002/tox.24382 (PMC11911904; doi:10.1002/tox.24382)
Supplement: Supplementary file 2 — Figure S2. [file TOX-40-509-s002.pdf]

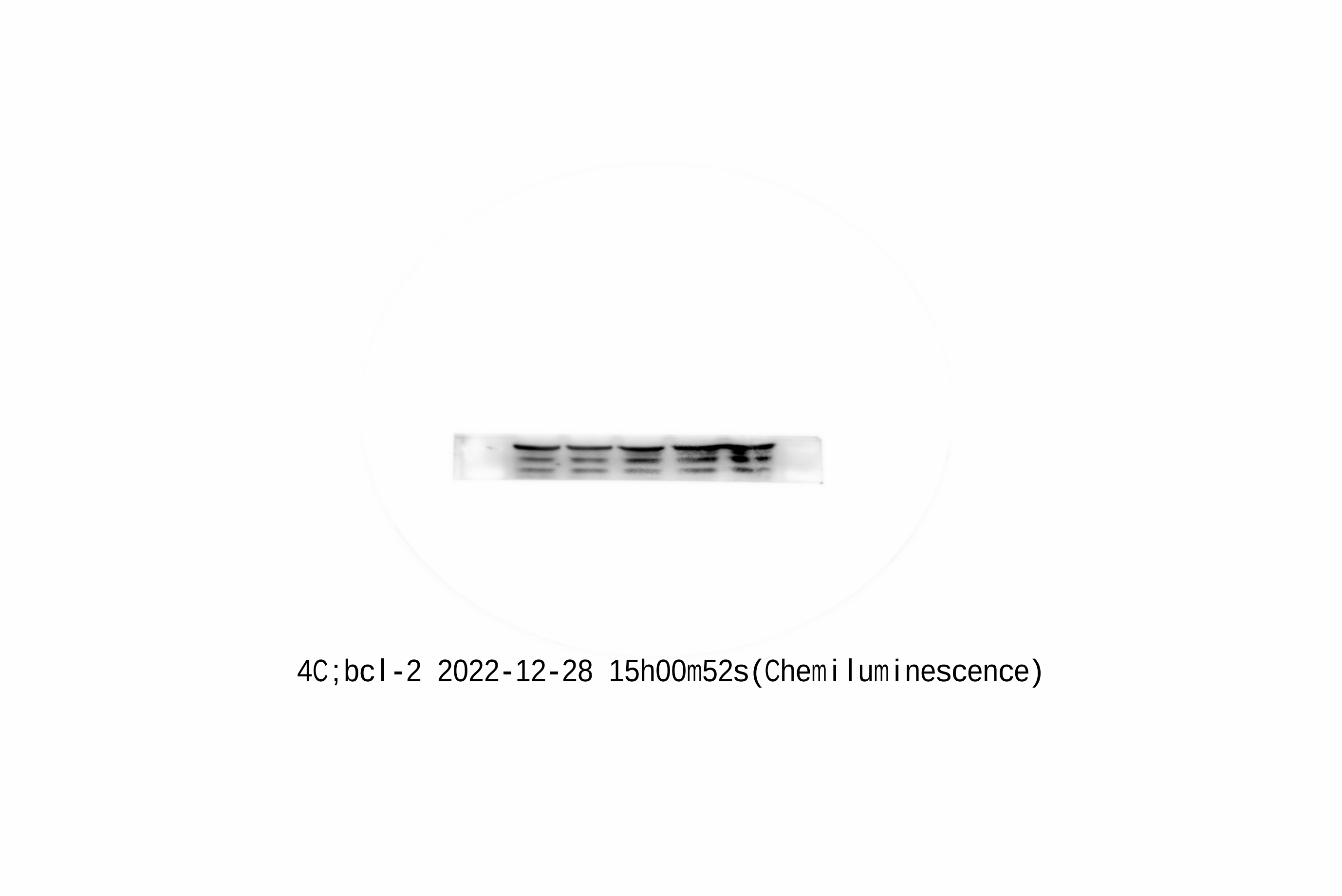
4C;bcl-2 2022-12-28 15h00m52s(Chemiluminescence)

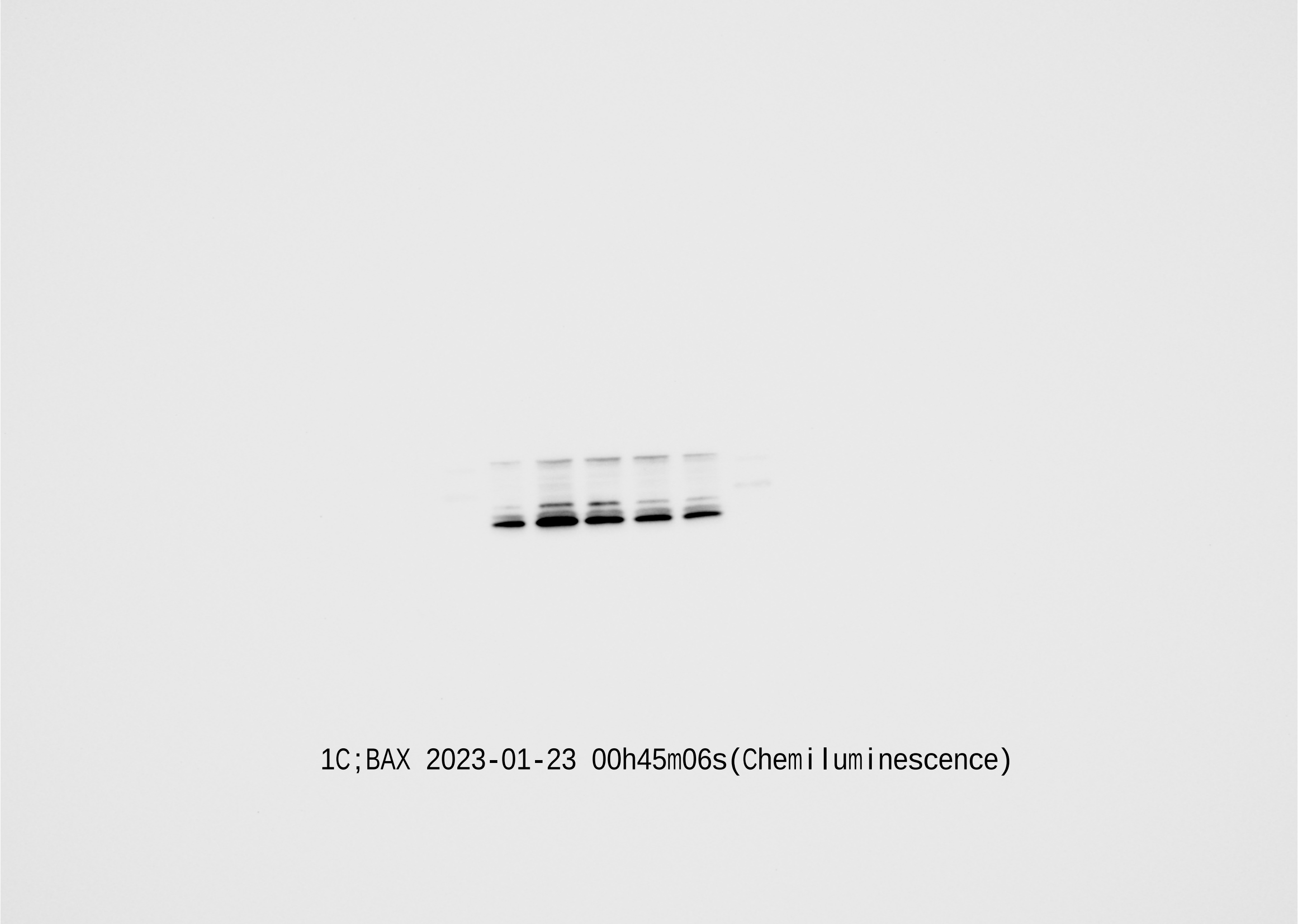
1C;BAX 2023-01-23 00h45m06s(Chemiluminescence)

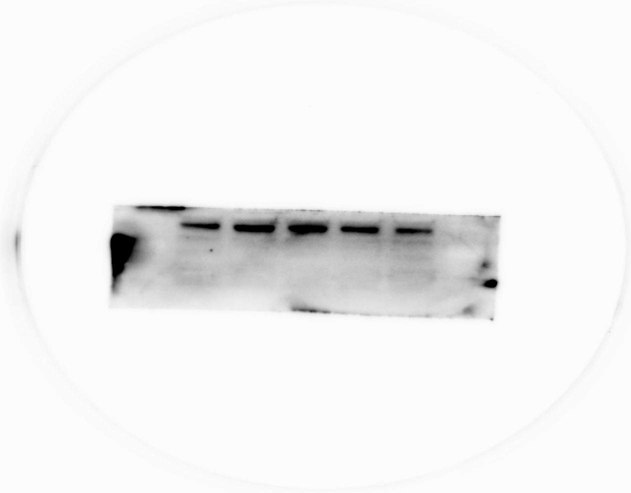

4B;Caspase-3 2023-01-02 13h13m20s(Chemiluminescence)

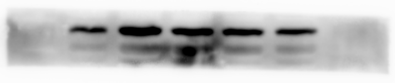

1B;P53 2023-01-23 00h22m04s(Chemiluminescence)

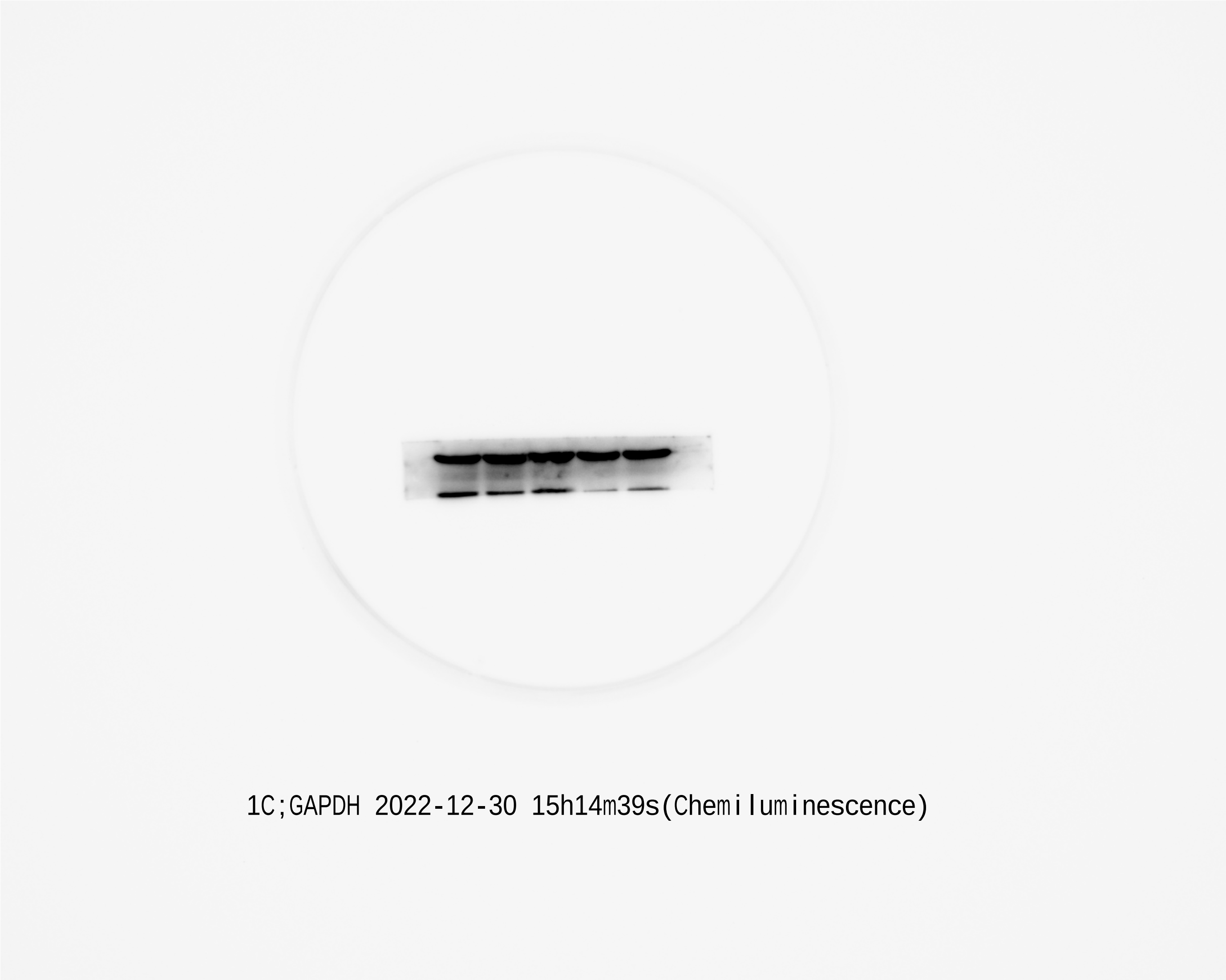

Figures S2
