# Supplementary material for: Combining Network Pharmacology and Experimental Verification to Ascertain the Mechanism of Action of Asparagus officinalis Against the Brain Damage Caused by Fluorosis
Source: Environ Toxicol. 2024 Jul 23;40(4):509–23. doi: 10.1002/tox.24382 (PMC11911904; doi:10.1002/tox.24382)
Supplement: Supplementary file 3 — Table S1. [file TOX-40-509-s001.docx]

| **Supplementary Table 1**. Target of active ingredient of Asparagine | |
| --- | --- |
| **Mol name** | **target gene** |
| beta-sitosterol | PGR、NCOA2、PTGS1、PTGS2、KCNH2、CHRM3、CHRM1、SCN5A、CHRM4、ADRA1A、CHRM2、ADRA1B、ADRB2、CHRNA2、SLC6A4、OPRM1、GABRA1、BCL2、BAX、CASP9、JUN、CASP3、CASP8、PRKCA、PON1、MAP2 |
| sitosterol | PGR、NCOA2、NR3C2 |
| pseudoprotodioscin_qt | PGR、NR3C2、NR3C1 |
| 7-Methoxy-2-methyl isoflavone | NOS2、PTGS1、CHRM3、CHRM1、ESR1、AR、ADRB1、SCN5A、PPARG、PTGS2、RXRA、ACHE、ADRA1B、SLC6A3、ADRB2、ADRA1D、SLC6A4、ESR2、GABRA1、MAPK14、GSK3B、LTA4H、MAOB、CHEK1、PRSS1、CCNA2、NCOA1、PKIA、CHRM5、OPRM1、NCOA2 |
| Stigmasterol | PGR、NR3C2、NCOA2、ADH1C、RXRA、NCOA1、PTGS1、PTGS2、ADRA2A、SLC6A2、SLC6A3、ADRB2、AKR1B1、PLAU、LTA4H、MAOB、MAOA、CTRB1、CHRM3、CHRM1、ADRB1、SCN5A、ADRA1A、CHRM2、ADRA1B、GABRA1 |
| diosgenin | PGR、NR3C2、RELA、AKT1、VEGFA、CDKN1A、TP63、PTGS2、FASN、SOD1、CAT、HIF1A、NR1I2、PLA2G4A、ABCC2、MTOR |
| quercetin | PTGS1、AR、PPARG、PTGS2、NCOA2、AKR1B1、PRSS1、KCNH2、SCN5A、ADRB2、MMP3、F7、RXRA、ACHE、GABRA1、MAOB、RELA、EGFR、AKT1、VEGFA、CCND1、BCL2、BCL2L1、FOS、CDKN1A、EIF6、BAX、CASP9、PLAU、MMP2、MMP9、MAPK1、IL10、EGF、RB1、TNFSF15、JUN、IL6、AHSA1、CASP3、TP63、ELK1、NFKBIA、POR、ODC1、CASP8、TOP1、RAF1、SOD1、PRKCA、MMP1、HIF1A、STAT1、RUNX1T1、ERBB2、PPARG、ACACA、HMOX1、CYP3A4、CYP1A2、CAV1、MYC、F3、GJA1、CYP1A1、ICAM1、IL1B、CCL2、SELE、VCAM1、PTGER3、CXCL8、PRKCB、BIRC5、DUOX2、NOS3、HSPB1、SULT1E1、MGAM、IL2、NR1I2、CYP1B1、CCNB1、PLAT、THBD、SERPINE1、COL1A1、IFNG、ALOX5、IL1A、MPO、TOP2A、NCF1、ABCG2、HAS2、GSTP1、NFE2L2、NQO1、PARP1、AHR、PSMD3、SLC2A4、COL3A1、CXCL11、CXCL2、DCAF5、NR1I3、CHEK2、INSR、CLDN4、PPARA、PPARD、HSF1、CRP、CXCL10、CHUK、SPP1、RUNX2、RASSF1、E2F1、E2F2、ACPP、CTSD、IGFBP3、IGF2、CD40LG、IRF1、ERBB3、PON1、DIO1、PCOLCE、NPEPPS、HK2、RASA1、GSTM1、GSTM2 |
